# Combinatorial metabolic engineering of *Streptomyces* sp. CB03234-S for the enhanced production of anthraquinone-fused enediyne tiancimycins

**DOI:** 10.1186/s12934-024-02399-w

**Published:** 2024-05-04

**Authors:** Zhoukang Zhuang, Wenping Kong, Zhongqing Wen, Nian Tong, Jing Lin, Fan Zhang, Zhiying Fan, Liwei Yi, Yong Huang, Yanwen Duan, Xiaohui Yan, Xiangcheng Zhu

**Affiliations:** 1 Xiangya International Academy of Translational Medicine, Central South University, Changsha, 410013 China; 2 The Affiliated Nanhua Hospital, Department of Pharmacy, Institute of Clinical Pharmacy, Hengyang Medical School, University of South China, Hengyang, 421002 China; 3 Hunan Engineering Research Center of Combinatorial Biosynthesis and Natural Product Drug Discovery, Changsha, 410011 China; 4 National Engineering Research Center of Combinatorial Biosynthesis for Drug Discovery, Changsha, 410013 China; 5 State Key Laboratory of Component-Based Chinese Medicine, Tianjin University of Traditional Chinese Medicine, Tianjin, 301617 China

**Keywords:** Combinatorial metabolic engineering, Titer improvement, Anthraquinone-fused enediynes, Genome reduction, Large-scale fermentation

## Abstract

**Background:**

Anthraquinone-fused enediynes (AFEs) are excellent payloads for antibody-drug conjugates (ADCs). The yields of AFEs in the original bacterial hosts are extremely low. Multiple traditional methods had been adopted to enhance the production of the AFEs. Despite these efforts, the production titers of these compounds are still low, presenting a practical challenge for their development. Tiancimycins (TNMs) are a class of AFEs produced by *Streptomyces* sp. CB03234. One of their salient features is that they exhibit rapid and complete cell killing ability against various cancer cell lines.

**Results:**

In this study, a combinatorial metabolic engineering strategy guided by the CB03234-S genome and transcriptome was employed to improve the titers of TNMs. First, re-sequencing of CB03234-S (Ribosome engineered mutant strains) genome revealed the deletion of a 583-kb DNA fragment, accounting for about 7.5% of its genome. Second, by individual or combined inactivation of seven potential precursor competitive biosynthetic gene clusters (BGCs) in CB03234-S, a double-BGC inactivation mutant, S1009, was identified with an improved TNMs titer of 28.2 ± 0.8 mg/L. Third, overexpression of five essential biosynthetic genes, including two post-modification genes, and three self-resistance auxiliary genes, was also conducted, through which we discovered that mutants carrying the core genes, *tnmE* or *tnmE10*, exhibited enhanced TNMs production. The average TNMs yield reached 43.5 ± 2.4 mg/L in a 30-L fermenter, representing an approximately 360% increase over CB03234-S and the highest titer among all AFEs to date. Moreover, the resulting mutant produced TNM-W, a unique TNM derivative with a double bond instead of a common ethylene oxide moiety. Preliminary studies suggested that TNM-W was probably converted from TNM-A by both TnmE and TnmE10.

**Conclusions:**

Based on the genome and transcriptome analyses, we adopted a combined metabolic engineering strategy for precursor enrichment and biosynthetic pathway reorganization to construct a high-yield strain of TNMs based on CB03234-S. Our study establishes a solid basis for the clinical development of AFE-based ADCs.

**Supplementary Information:**

The online version contains supplementary material available at 10.1186/s12934-024-02399-w.

## Introduction

**Fig. 1:**
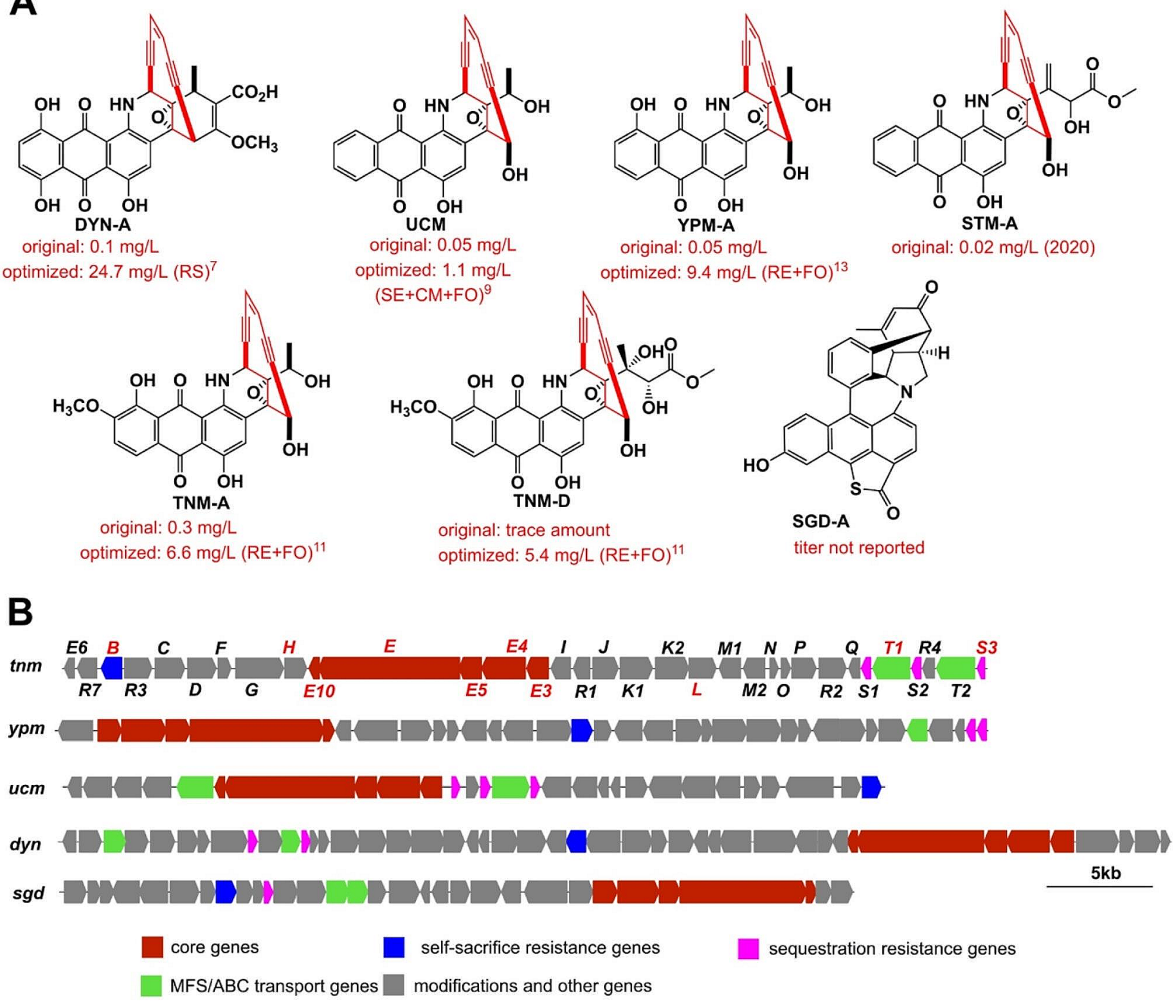
Overview of the anthraquinone-fused enediynes (AFEs). (**A**) The structures and reported titers of representative AFEs, including DYN-A, UCM, YPM-A, STM-A, TNM-A, TNM-D, and SGD-A. The methods used for improving the titers are indicated in bracket. RS: resin supplement, SE: strain engineering, CM: chemical mutagenesis, FO: fermentation optimization, RE: ribosome engineering. (**B**) The biosynthetic gene clusters (BGCs) of AFEs. Highly conserved core genes and possible self-resistance genes are marked in different colors

Enediyne natural products are among the most cytotoxic small molecules known to date. They are ideal payloads for the antibody-drug conjugates (ADCs) [1]. The anthraquinone-fused enediynes (AFEs) possess unique structural feature in which the anthraquinone moiety is fused with the 10-membered enediyne core to facilitate the interaction with DNA [2]. Six groups of AFEs have been identified to date, including dynemicins (DYNs), uncialamycin (UCM), tiancimycins (TNMs), yangpumicins (YPMs), sealutomicins (STMs), and non-canonical aromatized sungeidines (SGDs) (Fig. 1A). In recent preclinical studies, different synthetic UCM analogs have been applied as ADC payloads and exhibited potent antitumor activity. Some of the analogs displayed a strong bystander-killing effect, which is beneficial for their antitumor efficacy [3–5]. Furthermore, the potent antitumor effect of targeted TNM-A delivery via liposomes supports an alternative strategy for the translation of AFEs as nanomedicines [6]. However, the low production titers of AFEs severely limit their clinical development. Multiple traditional methods, such as resin supplement, ribosome engineering, fermentation optimization, and genome shuffling, had been adopted to enhance the production of the AFEs (Fig. 1A), such as DYNs [7, 8], UCM [9], TNMs [10–12], and YPMs [13]. Despite these efforts, the production titers of these compounds are still low, presenting a practical challenge for their development. Therefore, more rational strategies are required to break through the bottleneck in AFE production.

Compared with conventional strain improvement approaches, metabolic engineering strategy modifies host metabolism to direct metabolic flux into target pathway by overexpressing the genes involved in precursor supply and target product formation and eliminating competing pathways [14–16]. This strategy has been successfully applied to improve the yields of many antibiotics, such as the veterinary medicine salinomycin [17], the immunosuppressive agent FK506 [18], and the natural herbicide thaxtomin [19]. In the five biosynthetic gene clusters (BGCs) of AFEs (Fig. 1B), many encoded proteins are highly conserved [20, 21]. Recent studies on DYN biosynthesis have revealed the dual role of PKSE in the formation of both the 10-membered enediyne core and the anthraquinone moiety [22, 23]. The biosynthetic studies on AFEs suggests that they share a common pathway in the early steps, and their structural differences are attributed to different post-modification enzymes [21]. For example, the cytochrome P450 hydroxylase TnmL and the *O*-methyltransferase TnmH are essential for the introduction of the hydroxyl and methoxy groups on the A-ring of TNM-A and TNM-D [24]. The extreme toxicity of enediynes has led their producers to evolve different self-resistance mechanisms, including the self-sacrifice proteins [25, 26]. The latest investigation on the self-resistance mechanism to TNMs has revealed a family of sequestration proteins (TnmS1/S2/S3) that provide resistance to the host. Such a mechanism is generic to the AFEs because homologs of TnmS1/S2/S3 are present in all BGCs for AFEs [27]. In addition, the putative self-sacrifice protein TnmB and the putative drug efflux pump TnmT1 may also contribute to resistance to TNMs.

Based on our previous ribosome engineering mutant *Streptomyces* sp. CB03234-S [11], we adopted a combinatorial metabolic engineering strategy to further improve the titer of TNMs in this study. Using the genomic and transcriptomic data of CB03234-S, competitive BGCs were inactivated and their effects on TNM production were investigated. Furthermore, multiple biosynthetic genes, including *pksE* core genes, post-modification genes, and genes related to self-resistance, were overexpressed to obtain a high-producing strain for TNMs. Our work makes the large-scale production of TNMs practical and provides a solid basis for the future clinical development of AFEs as payloads for ADCs.

## Materials and methods

### Strains, plasmids, and growth conditions

*Streptomyces* sp. CB03234-S [11] was used in this study. The strains and plasmids used or constructed in this study, as well as all designed primers, are listed in Tables S1 and S2 (Supplementary Material). CB03234-S and related mutants were grown on Gauze’s medium (G1) at 30 °C for sporulation. Tryptic soy broth (TSB) was used as the seed medium. Optimal production (OP) medium (15 g/L soluble starch, 15 g/L yeast extract, 2 g/L CaCO_3_, 0.1 g/L CuSO_4_•5H_2_O, 5 mg/L NaI, 1% w/w Diaion HP20 resins) was used for liquid fermentation. *E. coil* DH5α was used for cloning and S17-1 was used for intergeneric conjugation with CB03234-S. Mannitol soya flour solid medium was used for intergeneric conjugation. Antibiotics (40 mg/L nalidixic acid, 50 mg/L apramycin, 60 mg/L streptomycin, 25 mg/L thiostrepton, or 50 mg/L kanamycin) were added as required. All common biological and chemical reagents were obtained from standard commercial sources.

### Genome sequencing and transcriptome analysis of CB03234-S

CB03234-S was cultivated in TSB medium at 30 °C for 36 h. Mycelia were collected, washed with ddH_2_O, and sent to Biomarker Technologies Corporation (Beijing, China) for genome sequencing on an Illumina Hiseq 2000 platform. High-quality clean reads were mapped onto the CB03234 reference genome (NCBI accession Number: NZ_LIYH00000000). The annotation of secondary metabolite BGCs in the sequenced genome of CB03234-S (Table S3) was performed using antiSMASH 5.0 software [28]. RNA sequencing data of CB03234 and CB03234-S from our previous study [29] were analyzed herein, and the raw sequencing reads were uploaded to the sequencing read archive (NCBI accession: PRJNA530700). Gene expression was normalized as fragments per kilobase of exon per million fragments mapped (FPKM). The expression level of the internal housekeeping gene *hrdB* (encoding the principal sigma factor of RNA polymerase) was used as a reference to normalize the expression of target genes from different competitive BGCs, and heat maps were drawn using Log2 (target gene/*hrdB*) transformed data.

### Inactivation of competitive BGCs in CB03234-S

Genetic manipulation of CB03234-S was carried out according to previously described procedures [30]. The core genes of seven competitive BGCs (Table S4) were deleted individually via homologous recombination. To construct a gene-knockout plasmid, regions approximately 2 kb upstream and downstream of the target gene, as well as the 810 bp thiostrepton resistance gene (*tsr*), were amplified using the high-fidelity Golden PCR Mix TSE101 (Tsingke Biotech. Co., Changsha, China). These DNA fragments were fused together and cloned into the *Hind*III/*Xba*I sites of pOJ260 using the Trelief SoSoo Cloning Kit (Tsingke). The resulting plasmid was verified by sequencing (Tsingke) and then introduced into CB03234-S via conjugation. The thiostrepton-resistant (Tsr^R^) and apramycin-sensitive (Apr^S^) exconjugants were verified using PCR amplification (Fig. S1) to obtain the BGC inactivation mutants, S1001‒S1007 (Table S1).

The same procedure was used to construct the double inactivation plasmids, but *tsr* was replaced with the kanamycin resistance gene (*kan*). The resulting plasmids were introduced into S1004 (Δ8-BGC) to generate S1008 (Δ2/8-BGCs) and S1009 (Δ15/Δ8-BGCs), which were both Apr^S^, Kan^R^, and Tsr^R^. The genotypes of the S1008 and S1009 mutants were confirmed using PCR verification (Fig. S1).

### Overexpression of target genes in CB03234-S and derivative mutants

The core genes *tnmE3/E4/E5/E/E10*, post-modification genes *tnmL/H*, and putative self-resistance genes *tnmB/T1/S3* were overexpressed in CB03234-S. Each target gene or gene combination was amplified and cloned into linearized pSET152 using *Hind*III/*Nde*I sites via seamless cloning. The constructed overexpression plasmids were respectively introduced into CB03234-S to generate mutants S1011‒S1018 (Table S1). The S1010 mutant carrying null pSET152 was also obtained as the negative control. Finally, *tnmE10*, *tnmE*, or *tnmE/E10* were further introduced into S1009 to generate mutants S1019‒S1021 (Table S1).

### Evaluating TNM-A tolerance of selected strains

To test the tolerance of each target strain to TNM-A, 1 mg/mL TNM-A stock solution was added to the medium to obtain G1 solid plates with different concentrations of TNM-A (0.5, 1.0, 2.0, 4.0, or 8.0 mg/L). The spore suspension of each strain was adjusted to 1.0 × 10^8^/mL, and then 100 µL spores were spread onto each G1 solid plates. After incubation at 30 °C for 3‒4 days, growth of each strain was observed and the number of single colonies was counted.

### Fermentation production and HPLC analysis of TNMs

Briefly, 50 µL of spore suspension was inoculated into 50 mL TSB medium and cultivated at 220 rpm and 30 °C for 36 h. Then, 3 mL (6% v/v) of each suspension was transferred into 50 mL of OP medium and cultivated under the same conditions for seven days. The collected mycelia and resins were ultrasonically treated with a total of 50 mL methanol. The combined extracts were subjected to high-performance liquid chromatography (HPLC) analysis on a Waters E2695 HPLC system equipped with a PDA detector and a Welch Ultimate AQ-C18 column (5 μm, 250 × 4.6 mm, Welch Materials Inc., Shanghai, China). The mobile phase consisted of A (H_2_O) and B (CH_3_CN) at a flow rate of 1 mL/min. A gradient program (90% A for 1 min; 90% A to 5% A for 17 min; 5% A for 2 min; 5% A to 90% A for 3 min, followed by 90% A for 2 min) was applied to detect TNM-A and TNM-D at 540 nm.

### Scaled-up production of TNMs in a 30 L fermenter

Scaled-up production of TNMs was performed in a T&J C-type 30 L fermenter (T&J Bioengineering Co., Ltd., Shanghai, China). Briefly, 50 µL spore suspension was inoculated into 50 ml TSB medium and cultured at 30 °C for 36 h. Subsequently, 5 mL primary seeds were inoculated into 500 mL TSB medium and cultured at 30 °C for an additional 24 h. Then 1.5 L of seeds was transferred into a 30-L fermenter containing 25 L of OP medium for the fermentation. The stirrer speed (100‒300 rpm), aeration rate (350 L/h), and air pressure (0.02‒0.04 MPa) were coordinated to maintain 40%∼60% dissolved oxygen (DO) in the fermentation broth. At the end of the exponential growth phase, the pH of the broth was adjusted to approximately 8.0 automatically by adding 50 g/L of acidic soluble starch solution (pH 1.0, adjusted using HCl). Samples were periodically collected and analyzed to monitor the yield of TNMs until the end of fermentation.

### Isolation, structural characterization, and cytotoxicity evaluation of TNM-W

The resins were recovered from 25 L fermentation broth containing S1021 using a 60-mesh stainless sieve filter (diameter 0.125 mm) and then ultrasonically extracted with 1 L methanol four times (10 min each). The combined extracts were concentrated to obtain a brown-black crude extract, which was then resuspended in 500 mL pure H_2_O and extracted again with 500 mL ethyl acetate (EA) three times. The EA extracts were evaporated, redissolved in 5 mL methanol, and purified using a CombiFlash RF200 preparative chromatography system (Teledyne ISCO, Lincoln, NE, USA) equipped with a Welch AQ-C18 flash column (20–40 μm, 80 g). Using a linear gradient from 10% methanol in H_2_O to 90% methanol in H_2_O at a flow rate of 10 mL/min, the fractions containing the target product TNM-W were combined and dried in vacuo. The resulting crude product was subjected to Sephadex LH-20 column chromatography by eluting with methanol. The collected fractions were further purified using semipreparative HPLC to yield 4.7 mg of pure TNM-W. The structure of TNM-W was characterized by spectral analyses, including high-resolution mass spectrometry (HR-MS), nuclear magnetic resonance (NMR), and infrared spectroscopy (IR).

To assess the cytotoxicity of TNM-W, the A549, KPL-4, Jurkat, and Caco-2 tumor cell lines were selected using TNM-A as a reference. Briefly, A549 and KPL-4 cells were cultured in DMEM, whereas Jurkat and Caco-2 cells were cultured in RPMI 1640. Each cell line was seeded in 96-well plates (4 × 10^3^ cells per well), cultivated for 24 h, and then treated with different concentrations (0.0001, 0.001, 0.01, 0.1, 1, 5, 10, or 50 nM) of TNM-W or TNM-A (100 µL media per well). After 72 h of incubation, cell viability was measured using a CCK-8 assay to determine the corresponding IC_50_ values. All experiments were carried out in triplicate.

### Statistical analysis

All fermentation experiments were performed at least in triplicate. At least three independent experiments were performed for each quantification. All data were statistically analyzed using GraphPad Primer 5.0 and presented as mean ± SD. Significant difference analysis was performed using Student’s *t*-test. The significance level was set at *P* < 0.05 (*P < 0.05 was significant difference, **P < 0.01 and ***P < 0.001 were highly significant difference). Two-group comparisons were performed using Student’s *t*-test. All tests utilized the two-tailed methodology.

## Results and discussion

### Genomic and transcriptomic analyses of CB03234-S

**Fig. 2:**
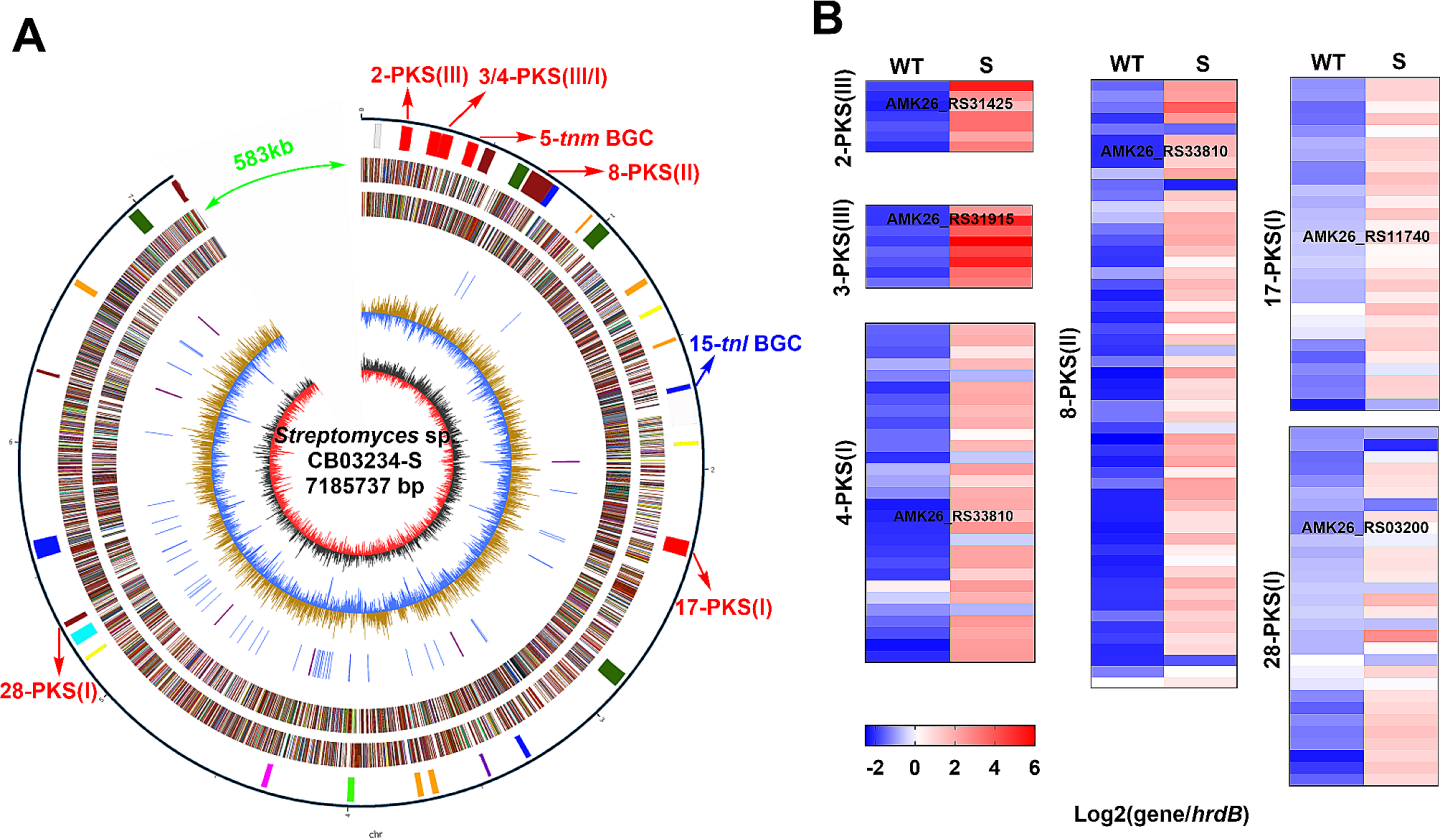
Genomic and transcriptomic analyses of CB03234-S. (**A**) Map of the CB03234-S genome. From the outside: Circle 1 displays the distribution of 32 BGCs, the 583 kb missing fragment (green), and different PKS BGCs, including *tnm* (red) and the known *tnl* terpene BGC (blue). Circles 2 and 3 (forward and reverse strands, respectively) show predicted protein-coding sequences in colors according to Clusters of Orthologous Gene function categories. Circles 4 and 5 (forward and reverse strands, respectively) show the distribution of essential genes (cell division and chromosome partitioning, replication, transcription, translation, amino acid/nucleotide transport, and metabolism). Circle 6 shows GC content. Circle 7 shows GC bias. (**B**) Heat maps of the six remaining PKS BGCs in CB03234 and CB03234-S. The gene bank number of the core *pks* gene in each PKS BGC was indicated. The core genes for each BGC are listed in Table S4. *HrdB* expression was used as an internal control

CB03234-S was obtained by ribosome engineering using streptomycin as the inducer. It could produce 6.6 mg/L of TNM-A and 5.4 mg/L of TNM-D (Fig. 1A) [12]. To identify possible genetic traits affecting the production of TNMs, the whole genome of CB03234-S was first sequenced. AntiSMASH analysis of CB03234 genome showed that it contains 39 putative BGCs, including the BGC for TNMs (#5-BGC), the BGC for tiancilactones (TNLs) (#15-BGC) [31], as well as other nine putative polyketide BGCs (Table S3). In contrast, in the genome of CB03234-S a large segment of 583,065 bp that covered seven BGCs, including three PKS BGCs (#33, #38, and #39), two lantipeptide BGCs, one amglyccycl BGC, and one terpene BGC, was missing. The missing nucleotides in CB03234-S represented a 7.5% reduction in the genome size of CB03234 (Fig. 2A and Table S3). Besides the *tnm* BGC, the products of the other six PKS BGCs in CB03234-S, including three Type I PKS BGCs (#4, #17, and #28), one Type II PKS BGC (#8), and two Type III PKSs (#2 and #3), have not been identified yet (Table S3). After analyzing the transcriptomic data from our previous study [29], we found that expression of most genes in the six remaining PKS BGCs, were up-regulated in CB03234-S in comparison with that in CB03234 (Fig. 2B and Tables S6‒S13). Genome reduction is an efficient strategy for constructing high-yield producers of secondary metabolites [32, 33]. The large-segment genomic reduction in CB03234-S provides an encouraging starting point for our following work.

### Inactivation of putative competitive BGCs enhances TNM production

Various biosynthetic pathways in the same *Streptomyces* strain are often competing for common precursors and cofactors [34, 35]. Therefore, metabolic engineering strategies, such as increasing the precursor pool and eliminating competing biosynthetic pathways, have been extensively applied to improve the titer of target natural products [36, 37]. Because acetyl-CoA and malonyl-CoA are the building blocks for TNM biosynthesis [22, 38], the six PKS BGCs other than *tnm* in CB03234-S may potentially compete for CoA precursors with the biosynthesis of TNM. On the other hand, the by-products TNLs are derived from the intermediate geranyl-geranyl pyrophosphate, which, in turn, is generated from pyruvate and glyceraldehyde-3-phosphate through the methylerythritol phosphate pathway [39]. Since both pyruvate and glyceraldehyde-3-phosphate are early precursors of acetyl-CoA, #15-*tnl* could also shunt the carbon flux of the CoA precursors and energy source. Based on the above deductions, seven BGCs were inactivated in CB03234-S to produce mutants S1001 to S1007 (Fig. S1), which were subsequently evaluated for their possible influence on the production of TNMs.

**Fig. 3:**
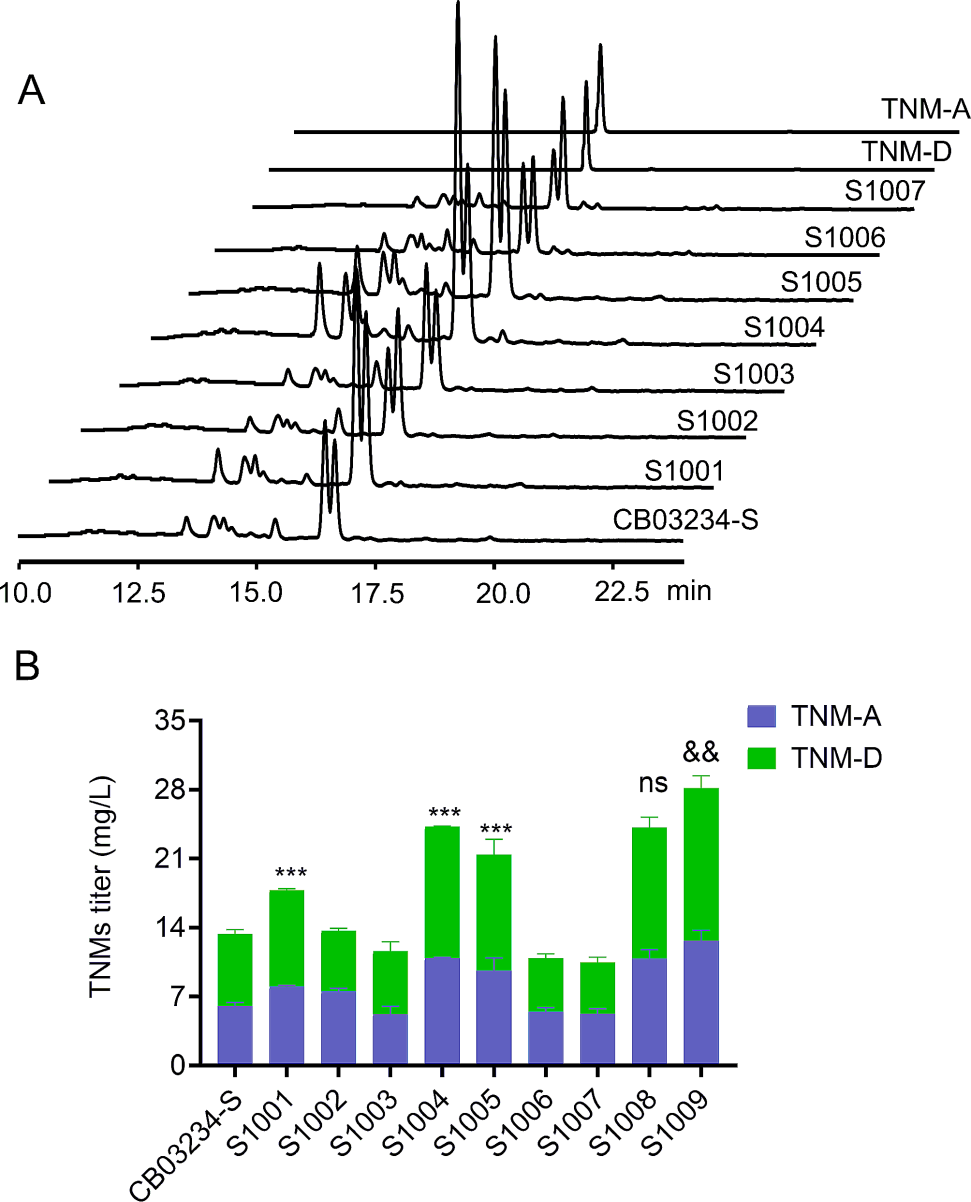
Comparisons of CB03234-S and derived mutants after seven days of fermentation in OP medium. (**A**) HPLC profiles of CB03234-S and derived mutants at 540 nm. (**B**) Average TNMs titers from single BGC inactivation mutants, S1001 (Δ#2-BGC), S1002 (Δ#3-BGC), S1003 (Δ#4-BGC), S1004 (Δ#8-BGC), S1005 (Δ#15-BGC), S1006 (Δ#17-BGC), and S1007 (Δ#28-BGC), and double BGC inactivation mutants (S1008 (Δ#2/8-BGC) and S1009 (Δ#15/8-BGC). ***P < 0.001 vs. CB03234-S; ^&&^*P* < 0.01 vs. S1004; ns: *P* > 0.05 vs. S1004; sample size, *n* = 3

The fermentation results indicated that only the inactivation of #2-BGC (S1001), #8-BGC (S1004), and #15-BGC (S1005) enhanced the production of TNMs. In contrast, silencing of the other four BGCs had no apparent effect (Fig. 3A). Compared with that in CB03234-S (12.0 mg/L), the TNMs titers in S1001, S1004, and S1005 were 18.3 ± 1.1 mg/L, 24.5 ± 1.1 mg/L, and 21.3 ± 2.3 mg/L, respectively (Fig. 3B); S1004 showed the most significant titer improvement of 90%. By comparing the HPLC profiles of the mutants and CB03234-S, an unknown metabolite, **1**, was found to correlate with #8-BGC (Fig. S2A). Although no distinct novel metabolite was observed in S1001, overexpression of the core *pks* gene (AMK26_RS31425) from #2-BGC generated a new peak, **2** (Fig. S2B). We have previously characterized the product of #15-BGC as tiancilactone, a diterpene with chloroanthranilate and γ-butyrolactone moieties [31]. We proposed that the inactivation of BGCs #2, #8, and #15 may direct the carbon flux towards the biosynthesis of TNMs, thus resulting in titer improvement.

Next, the double BGC inactivation mutants, S1008 (Δ#2/#8-BGCs) and S1009 (Δ#15/#8-BGCs), derived from S1004, were constructed and evaluated. S1008 did not exhibit an increase in TNM titer over S1004 (Fig. 3B), probably due to the redundant inactivation of similar PKS competing pathways [17]. In contrast, the TNMs titer in S1009 increased to 28.2 ± 0.8 mg/L, representing approximately a 230% enhancement (Fig. 3B); this is a logical outcome because #15-BGC involves different precursors and metabolic pathways than PKS BGCs. Collectively, these data provided new insights for titer improvement via the manipulation of disparate competitive BGCs.

### Overexpression of key biosynthetic and auxiliary genes enhances TNMs production

Key biosynthetic genes often govern rate-limiting steps, such as the formation of a molecular scaffold or post-modification of critical intermediates, and thus have crucial impacts on antibiotic production [40]. In addition, because excessive accumulation of antibiotics can affect the growth of host cells or cause feedback inhibition of their biosynthesis, auxiliary genes responsible for self-resistance also play essential roles in supporting the production of antibiotics [41]. As such, the overexpression of corresponding rate-limiting genes or self-resistance genes has already been adopted to improve antibiotic production [42, 43].

**Fig. 4:**
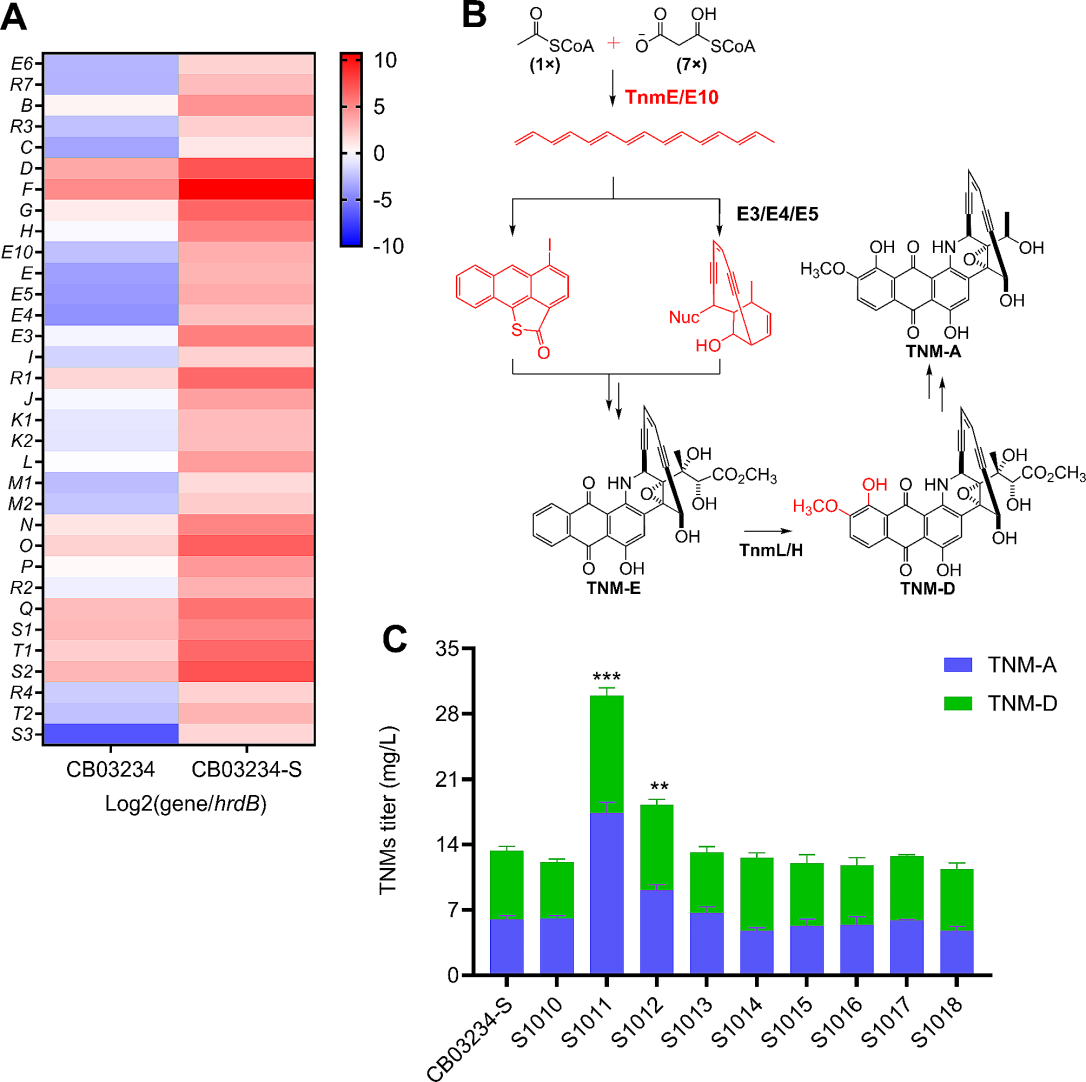
Overexpression of selected *tnm* genes for TNMs titer improvement. (**A**) Transcriptional heat map of *tnm* gene expression in CB03234-S and CB03234. (**B**) Roles of selected key biosynthetic genes (red) in the production of TNMs. (**C**) Comparison of TNMs titers from CB03234-S, S1010 (CB03234-S + pSET152), and related mutants, including those overexpressing key biosynthetic genes: S1011 (*tnmE*), S1012 (*tnmE10*), S1013 (*tnmE3/E4/E5*), S1014 (*tnmH*), and S1015 (*tnmL*), and auxiliary genes (S1016 (*tnmT1*), S1017 (*tnmB*), and S1018 (*tnmS3*)). TNMs (TNM-A and TNM-D). **P < 0.01 vs. CB03234-S, ***P < 0.001 vs. CB03234-S; sample size, *n* = 3

Based on transcriptomic data, the expression levels of all 34 genes in *tnm* were up-regulated in CB03234-S to varying degrees (Fig. 4A and Table S5), consistent with the increased production of TNMs in this strain. Among these biosynthetic genes, the highly conserved minimal *pksE* cassette (*tnmE3/E4/E5/E/E10*) is indispensable for the skeleton formation of AFEs, but the exact roles of TnmE3/E4/E5 are still uncertain [44]. The TnmL and TnmH are responsible for the post-modification of TNM intermediates to generate the final products TNM-D and TNM-A [24] (Fig. 4B), whereas TnmS1/S2/S3 have been reported to be important for the sequestration of TNM-A [27]. The *tnmB* encodes an enediyne self-sacrifice protein homologous to CalU16 (79% identity and 85% similarity), a known enediyne self-resistance protein [25, 26], and *tnmT1* encodes a major facilitator superfamily (MFS) transporter that may serve as an efflux pump for the exportation of intracellular TNMs. These auxiliary genes may contribute to host self-resistance to TNMs.

The above biosynthetic and auxiliary *tnm* genes were overexpressed in CB03234-S to generate the following mutants: S1011 (*tnmE*), S1012 (*tnmE10*), S1013 (*tnmE3/E4/E5*), S1014 (*tnmH*), S1015 (*tnmL*), S1016 (*tnmT1*), S1017 (*tnmB*), and S1018 (*tnmS3*). Our fermentation results indicated that only S1011 and S1012 exhibited enhanced production of TNMs, with titers of 27.7 ± 2.9 mg/L (120% improvement) and 18.5 ± 1.5 mg/L (40% improvement), respectively (Fig. 4C). Because PKSE is essential for the formation of both the enediyne core and anthraquinone moiety of AFEs [22], overexpression of *tnmE* could accelerate these key rate-limiting steps, thus improving the biosynthesis of TNMs. Previous groups have reported that type II thioesterase is responsible for the release of polyketide intermediates from PKS and the error correction of misloaded substrates or abnormal intermediates [45, 46]; thus, overexpression of *tnmE10* would likely increase the efficiency of TnmE and facilitate the production of TNMs. In comparison, overexpression of the other biosynthetic genes was ineffective, suggesting either their lack of involvement in rate-limiting steps or that their expression levels were already sufficiently high in CB03234-S. Overexpression of the three self-resistance auxiliary genes also showed no distinct effect on TNM titer (Fig. 4C); however, the corresponding mutants exhibited different degrees of heightened resistance to TNM-A on solid G1 plates (Fig. S3), clearly demonstrating the resistance function of these auxiliary genes. In our previous study, we discovered that the addition of macroporous resins during liquid fermentation promptly adsorbed extracellular TNMs to attenuate their toxic effects on host cells, thereby enhancing the production of TNMs [11]. Thus, we concluded that the detoxicating effect of resins far surpassed the self-resistance influences of auxiliary genes and covered the potential contributions of self-resistance genes to the titer improvement of TNMs.

### Construction of the final high-yielding strain and scaled-up validation

**Fig. 5:**
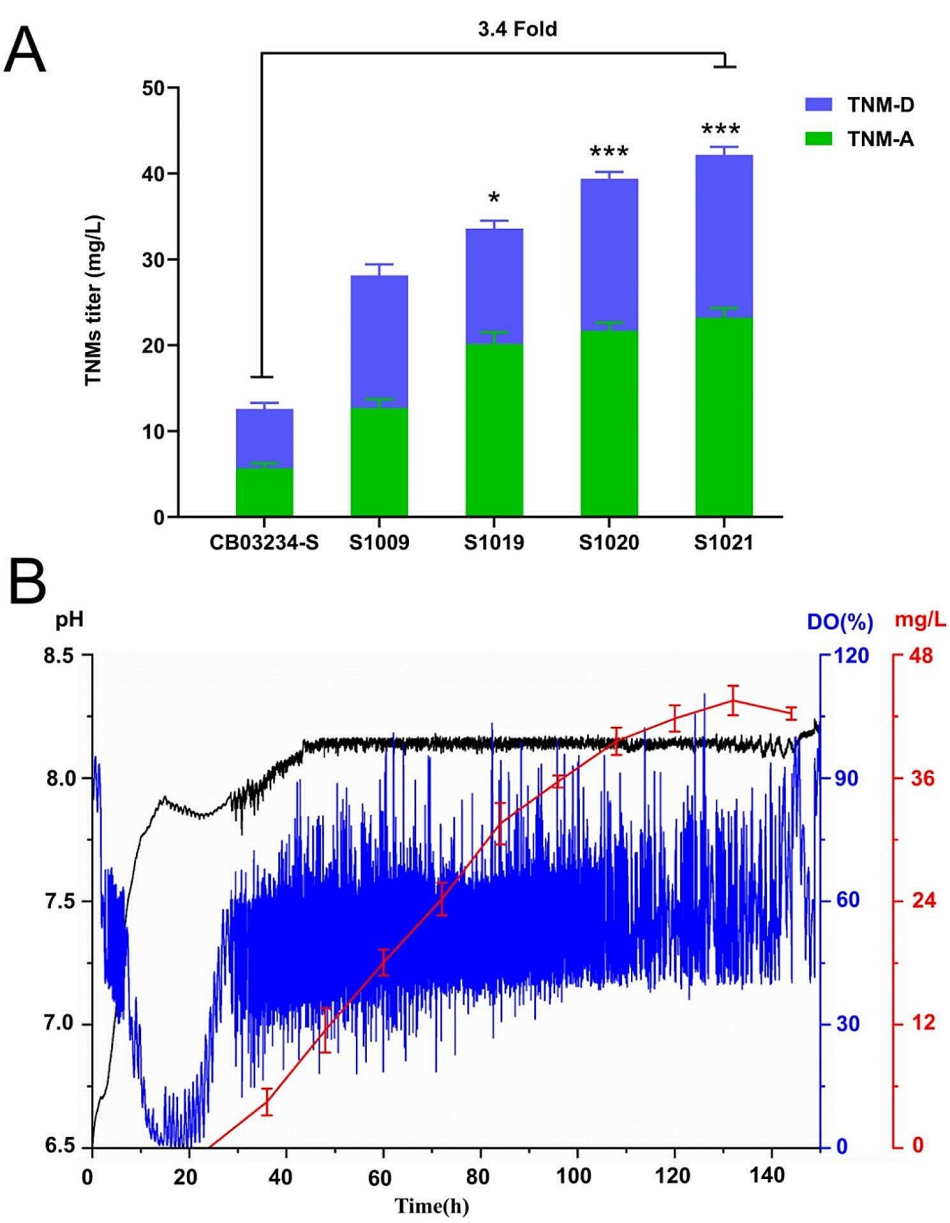
Construction, screening, and validation of the final high-yielding strain. (**A**) Comparison of TNMs titers from CB03234-S, S1009 (CB03224-S with BGC #8 and the *tnl* gene cluster inactivated), S1019 (S1009 + pSET-*tnmE10*), S1020 (S1009 + pSET-*tnmE*), and S1021 (S1009 + pSET-*tnmE/E10*). *P < 0.05 vs. S1009, ***P < 0.001 vs. S1009; sample size, *n* = 3. (**B**) Fermentation profiles of S1021 in a 30 L fermenter. Samples were collected every 12 h after initial 24-hour cultivation. DO (dissolved oxygen) and pH are detected online every minute

Overexpression of *tnmE10*, *tnmE*, and *tnmE/E10* was integrated into the double-BGC inactivation mutant S1009 to further enhance TNM titers, giving rise to the S1019 (*tnmE10*^S1009^), S1020 (*tnmE*^S1009^), and S1021 (*tnmE/E10*^S1009^) mutants. The highest TNM titer was achieved in S1021 (41.5 ± 2.7 mg/L in total, 23.2 ± 1.3 mg/L for TNM-A and 18.9 ± 2.1 mg/L for TNM-D), representing 340% titer improvement compared with that in CB03234-S (Fig. 5A). Thus, S1021 represents the final high-yielding strain produced through the combinatorial metabolic engineering of CB03234-S. Notably, the effect of each mutation on the production of TNMs in S1021 was not simply superposed. Such phenomena seem common in *Streptomycetes* owing to the complexity of their metabolic network [47], which also heralds the possibility of further improving the titer of TNMs by exploring other limiting metabolic factors.

Subsequently, S1021 was subjected to scaled-up validation in a 30 L fermenter. By adopting the previously established pH-correlation fed-batch strategy [11], the fermentation environment was kept relatively stable from the exponential growth phase. The concentration of TNMs steadily increased until the end of the fermentation (Fig. 5B). The average titer of TNMs from three separate batches was 43.5 ± 2.4 mg/L (the yields of TNM-A and TNM-D are 32.8 ± 1.9 mg/L and 10.7 ± 1.3 mg/L respectively). This represents the highest reported titer of AFEs to date.

### Discovery and characterization of a novel unnatural AFE analog, TNM-W

**Fig. 6:**
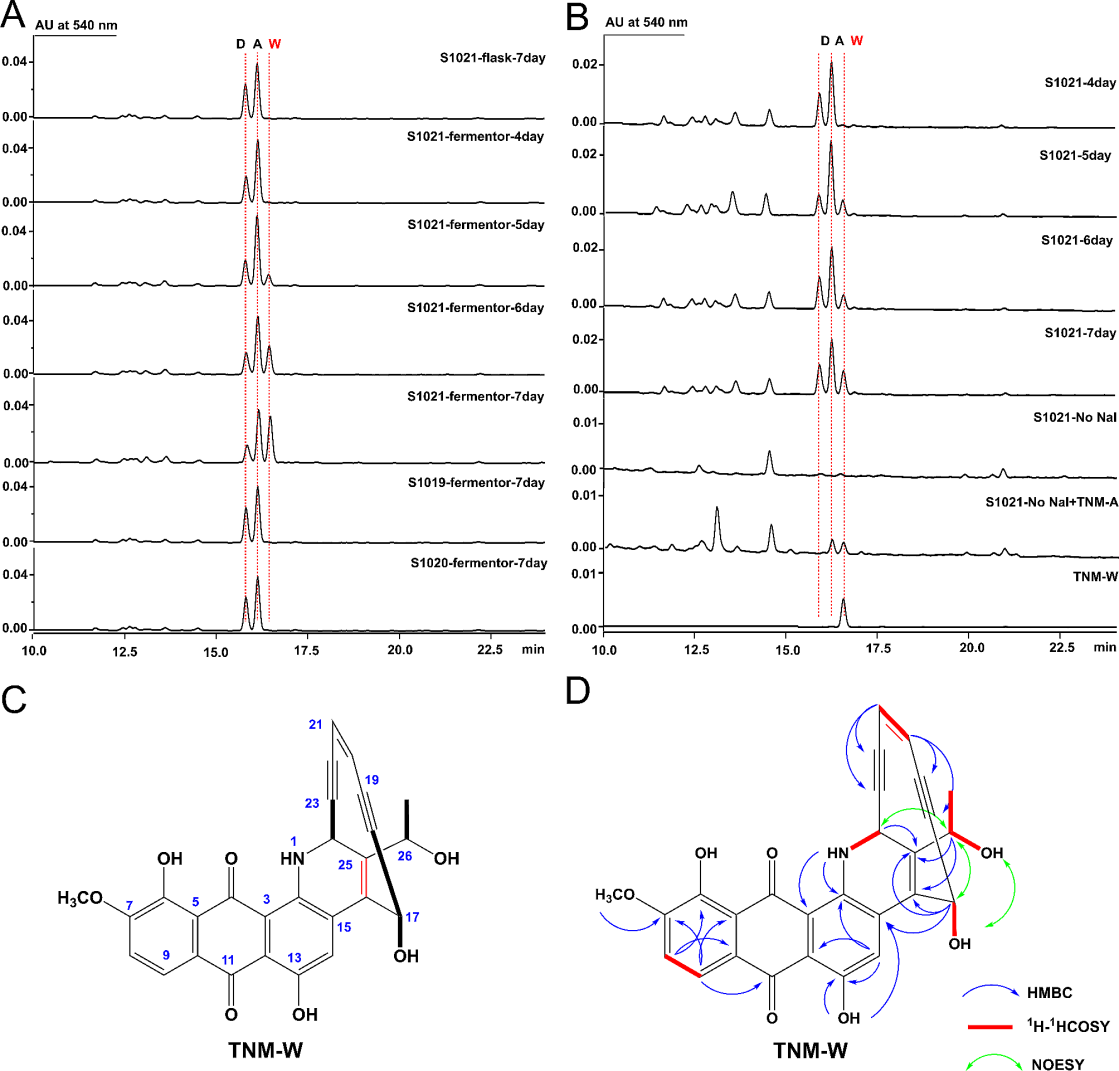
Discovery and characterization of a novel TNM analog, TNM-W. (**A**) Comparison of HPLC profiles for the production of TNM-A (A), TNM-D (D), and TNM-W (W) from S1021 in shaking flasks or 30 L fermenters, as well as S1019 and S1020 in 30 L fermenters. (**B**) HPLC profiles of S1021 in shaking flasks with pH-controlled OP medium. Samples were collected at different fermentation times or from OP medium without NaI with or without 50 µL of 1 mg/L TNM-A. S1021 is a mutant strain of CB03234-S in which BGC #8 and #15 were inactivated and *tnmE/E10* were overexpressed. (**C**) Structure of TNM-W. (**D**) Key ^1^H-^1^H correlation spectroscopy (COSY), nuclear overhauser effect spectroscopy (NOESY), and heteronuclear multiple bond correlation (HMBC) analysis of TNM-W.

During the scaled-up fermentation of S1021, a novel TNM analog, named TNM-W, appeared in the HPLC profile in addition to TNM-A/D (Fig. 6A). Time course monitoring revealed that TNM-W was detected after 5 days of S1021 fermentation in a 30-L fermenter. Its accumulation was negatively correlated with the concentration of TNM-A in a time-dependent manner (Fig. 6A and S4). To clarify the possible origin of TNM-W, scaled-up fermentation of S1019 and S1020 was also performed. However, neither strain generated TNM-W (Fig. 6A). According to the genotypes of S1019, S1020, and S1021, we hypothesized that the generation of TNM-W was dependent on both TnmE and TnmE10. Interestingly, S1021 produced TNM-W in the 30 L fermenter but not when fermented in a shaking flask (Fig. 6A); this may be a consequence of different pH, as the changes in pH were quite different in these two fermentation conditions. To test this hypothesis, pH-controlled fermentation of S1021 was performed in shaking flasks. When the pH of broth in the shaking flasks was maintained between 8.1 and 8.4 using the acidic starch solution, TNM-W was detected similarly as in the 30-L fermenter (Fig. 6B). In addition, direct bioconversion from exogenous TNM-A to TNM-W was performed under the same conditions. Without iodide, an essential element for the formation of the key intermediate iodoanthracene and subsequent biosynthesis of AFEs [23], neither TNMs nor TNM-W were detected in the OP medium. In this context, externally supplemented TNM-A was transformed into TNM-W by S1021 (Fig. 6B). Hence, we proposed that the artificial enzymatic complex TnmE/E10 can convert TNM-A into TNM-W under comparably stable fermentation conditions, especially pH. However, the specific mechanism remains to be further explored.

Pure TNM-W was successfully obtained through a series of isolation and purification steps. The molecular formula of TNM-W was determined to be C_27_H_20_NO_7_ using high-resolution electrospray ionization (*m/z* = 470.1240 [M + H]^+^, calculated for 470.1240) (Fig. S4). Spectral characterization and comparison identified that TNM-W only varied from TNM-A at the C-16 and C-25 positions (^13^C NMR signals *δ*_C−16_ = 124.9, *δ*_C−25_ = 138.4; key heteronuclear multiple bond correlation signals H-17 to C-16/C-25, H-26 to C-16/C-25, H-24 to C-25; Fig. S6-S11 and Table S5), at which the ethylene oxide moiety in TNM-A was replaced by a carbon-carbon double bond in TNM-W (Fig. 6C and D). The stereochemistry of TNM-W was assigned as 26*R* based on the ROESY correlations between H-24 and H-26 (Fig. 6D and Fig. S11). To date, all existing AFEs contain an epoxy ring unit (Fig. 1A), whose opening is supposed to trigger the Bergman cyclization of the enediyne core [48] to facilitate their bioactivities. TNM-W is the first unnatural AFE analog with a unique double bond instead of a common epoxy ring. TNM-W may be a self-detoxified side product that limits the further accumulation of highly toxic TNMs.

## Conclusions

Based on genomic and transcriptomic analyses, as well as an understanding of the biosynthesis of TNMs, a combinatorial metabolic engineering strategy was employed to reconstruct CB03234-S for further TNMs titer improvement in this study. Through the inactivation of potentially competitive BGCs, three competitive BGCs that were identified; however, only the successive inactivation of two BGCs with distinct pathways continuously improved the production of TNMs. Meanwhile, the overexpression of key biosynthetic and auxiliary genes revealed that *tnmE* and *tnmE10* govern the formation of both the enediyne core and anthraquinone moiety, which are important for strengthening the production of TNMs. After integrating these beneficial mutations, the high-yielding strain S1021 was constructed. An average yield of over 43 mg/L TNMs was achieved in a pilot-scale 30 L fermenter, representing the highest reported AFE titer to date. In addition, S1021 produced a novel TNM analog, TNM-W, which is the first unnatural AFE analog with a unique double bond in place of the common ethylene oxide moiety found in other AFEs. Our study establishes a solid basis for the industrial production of TNMs and provides a platform to enhance the production of other AFEs.

### Electronic supplementary material

Below is the link to the electronic supplementary material.

Supplementary Material 1

## Data Availability

No datasets were generated or analysed during the current study.
