# Supplementary material for: Combinatorial metabolic engineering of Streptomyces sp. CB03234-S for the enhanced production of anthraquinone-fused enediyne tiancimycins
Source: Microb Cell Fact. 2024 May 4;23:128. doi: 10.1186/s12934-024-02399-w (PMC11069151; doi:10.1186/s12934-024-02399-w)
Supplement: Supplementary file 1 — Supplementary Material 1 [file 12934_2024_2399_MOESM1_ESM.doc]

**Supplementary Material**

**Combinatorial metabolic engineering of *Streptomyces* sp. CB03234-S for the enhanced production of anthraquinone-fused enediyne tiancimycins**

Zhoukang Zhuang ^1,✝^, Wenping Kong ^1,✝^, Zhongqing Wen ^1^, Nian Tong ^1^, Jing Lin ^1^, Fan Zhang ^1^, Zhiying Fan ^1^, Liwei Yi ^1, 2^, Yong Huang ^1, 3^, Yanwen Duan ^*1, 3, 4^, Xiaohui Yan ^*1, 5^, Xiangcheng Zhu ^*1, 3, 4^

^1^Xiangya International Academy of Translational Medicine, Central South University, Changsha 410013, China

^2^The Affiliated Nanhua Hospital, Department of Pharmacy, Institute of Clinical Pharmacy, Hengyang Medical School, University of South China, Hengyang 421002, China

^3^Hunan Engineering Research Center of Combinatorial Biosynthesis and Natural Product Drug Discovery, Changsha 410011, China

^4^National Engineering Research Center of Combinatorial Biosynthesis for Drug Discovery, Changsha 410013, China

^5^State Key Laboratory of Component-Based Chinese Medicine, Tianjin University of Traditional Chinese Medicine, Tianjin 301617, China

^✝^Zhoukang Zhuang and Wenping Kong contributed equally to this work.

^*^To whom correspondence should be addressed: Central South University, Tongzipo Road，#172, Yuelu District, Changsha, Hunan 410013, China. Tel：(86) 731 8265 0539；Fax：(86) 731 8265 0551；Prof. Yanwen Duan, Email: ywduan66@sina.com, Prof. Xiangcheng Zhu, Email：[seanzhu1996@aliyun.com](mailto:seanzhu1996@aliyun.com), or Prof. Xiaohui Yan, Email: yanxh@tjutcm.edu.cn.

**Contents**

[**Table S1:** Strains and plasmids used in this study 3](#__RefHeading___Toc163714436)

[**Table S2:** Primers used in this study 5](#__RefHeading___Toc163714437)

[**Table S3:** Analysis of secondary metabolite BGCs in *S*. sp. CB03234 and CB03234-S 8](#__RefHeading___Toc163714438)

[**Table S4:** Knockout target genes and functions in biosynthetic gene clusters. 9](#__RefHeading___Toc163714439)

[**Table S5:** ^1^H and ^13^C NMR Spectroscopic Data (600 MHz, DMSO-*d*_6_) for TNM-W 10](#__RefHeading___Toc163714440)

[**Table S6:** Predicted functions of ORFs in #2-BGC and their average values derived from transcriptome analysis. 11](#__RefHeading___Toc163714441)

[**Table S7:** Predicted functions of ORFs in #3-BGC and their average values derived from transcriptome analysis. 12](#__RefHeading___Toc163714442)

[**Table S8:** Predicted functions of ORFs in #4-BGC and their average values derived from transcriptome analysis. 13](#__RefHeading___Toc163714443)

[**Table S9:** Predicted functions of ORFs in #5 (TNM)-BGC and their average values derived from transcriptome analysis. 14](#__RefHeading___Toc163714444)

[**Table S10:** Predicted functions of ORFs in #8-BGC and their average values derived from transcriptome analysis. 16](#__RefHeading___Toc163714445)

[**Table S11:** Predicted functions of ORFs in #15 (TNL)-BGC and their average values derived from transcriptome analysis. 18](#__RefHeading___Toc163714446)

[**Table S12:** Predicted functions of ORFs in #17-BGC and their average values derived from transcriptome analysis. 19](#__RefHeading___Toc163714447)

[**Table S13:** Predicted functions of ORFs in #28-BGC and their average values derived from transcriptome analysis. 20](#__RefHeading___Toc163714448)

[**Fig. S1.** Disruption of BGCs via PCR-targeting. (A) Schematic representation for disruption of BGC. (B) PCR analyses of the WT strain and the BGC double-cross mutant carried out using the primers listed in Table S2. S: CB03234-S; M: DL5000. 22](#__RefHeading___Toc163714449)

[**Fig. S2.** The HPLC profiles of the CB03234-S and mutant strains: (A) In the resin, HPLC profiles of CB03234-S and mutant strains, S1004 (Δ#8-BGC), S1005 (Δ#15-BGC); (B) In the supernatant, HPLC profiles of CB03234-S and mutant strains, CB03234-S-*AMK26*_*RS31425*, (overexpression of the core pks gene (AMK26_ RS31425) from #2-BGC in CB03234-S). 23](#__RefHeading___Toc163714450)

[**Fig. S3.** The resistance of overexpression resistance genes mutants to TNM-A; S1016 (CB03234-S+*tnmT1*); S1017 (CB03234-S+*tnmB*); S1018 (CB03234-S+*tnmS3*). 24](#__RefHeading___Toc163714451)

[**Fig. S4.** Time course of TNM-W, TNM-A, and TNM-D production of S1021 strain in 30 L fermenter. 25](#__RefHeading___Toc163714452)

[**Fig. S5.** HR-ESI (+) MS spectrum of TNM-W 26](#__RefHeading___Toc163714453)

[**Fig. S6.** ^1^H NMR (600 MHz) spectrum of TNM-W in DMSO-*d*_6_ 27](#__RefHeading___Toc163714454)

[**Fig. S7**. ^13^C NMR (150 MHz) spectrum of TNM-W in DMSO-*d*_6_ 28](#__RefHeading___Toc163714455)

[**Fig. S8**. HSQC NMR (600 MHz) spectrum of TNM-W in DMSO-*d*_6_ 29](#__RefHeading___Toc163714456)

[**Fig. S9**. ^1^H -^1^H COSY NMR (600 MHz) spectrum of TNM-W in DMSO-*d*_6_ 30](#__RefHeading___Toc163714457)

[**Fig. S10**. HMBC NMR (600 MHz) spectrum of TNM-W in DMSO-*d*_6_ 31](#__RefHeading___Toc163714458)

[**Fig. S11**. NOESY NMR (600 MHz) spectrum of TNM-W in DMSO-*d*_6_ 32](#__RefHeading___Toc163714459)

[**References** 32](#__RefHeading___Toc163714460)

**Table S1:** Strains and plasmids used in this study

| Strains or plasmid | Description | Sources |
| --- | --- | --- |
| *E. coli* |  |  |
| DH5α | *E. coli* host for plasmid construction | Commercial |
| S17-1 | *E. coli* host for intergeneric conjugation | Commercial |
| Strains |  |  |
| *S.* sp*.* CB03234 | Wild type strain of TNMs | (Yan et al., 2016) |
| CB03234-S | High-yielding mutant strain of TNMs | (Zhuang et al., 2019) |
| S1001 | Inactivation of PKS-2 in CB03234-S (Tsr^R^) | This work |
| S1002 | Inactivation of PKS-3 in CB03234-S (Tsr^R^) | This work |
| S1003 | Inactivation of PKS-4 in CB03234-S (Tsr^R^) | This work |
| S1004 | Inactivation of PKS-8 in CB03234-S (Tsr^R^) | This work |
| S1005 | Inactivation of TNL-15 in CB03234-S(Tsr^R^) | This work |
| S1006 | Inactivation of PKS-17 in CB03234-S (Tsr^R^) | This work |
| S1007 | Inactivation of PKS-28 in CB03234-S (Tsr^R^) | This work |
| S1008 | Mutant with PKS-2 and PKS-8 inactivated in CB03234-S (Tsr^R^ Kan^R^) | This work |
| S1009 | Mutant with PKS-8 and TNL-15 inactivated in CB03234-S (Tsr^R^ Kan^R^) | This work |
| S1010 | CB03234-S carrying pSET152 (Apr^R^) | (Huang et al., 2019) |
| S1011 | CB03234-S carrying p152-*tnmE* (Apr^R^) | This work |
| S1012 | CB03234-S carrying p152-*tnmE10* (Apr^R^) | This work |
| S1013 | CB03234-S carrying p152-*tnmE345* (Apr^R^) | This work |
| S1014 | CB03234-S carrying p152-*tnmL* (Apr^R^) | This work |
| S1015 | CB03234-S carrying p152-*tnmH* (Apr^R^) | This work |
| S1016 | CB03234-S carrying p152-*tnmT1* (Apr^R^) | This work |
| S1017 | CB03234-S carrying p152-*tnmB* (Apr^R^) | This work |
| S1018 | CB03234-S carrying p152-*tnmS3* (Apr^R^) | This work |
| S1019 | PKS-8 and TNL-15 inactivated with carrying p152*-tnmE10* in CB03234-S (Tsr^R^ Kan^R^ Apr^R^) | This work |
| S1020 | PKS-8 and TNL-15 inactivated with carrying p152*-tnmE* in CB03234-S (Tsr^R^ Kan^R^ Apr^R^) | This work |
| S1021 | PKS-8 and TNL-15 inactivated carrying p152*-tnmE-E10* in CB03234-S (Tsr^R^ Kan^R^ Apr^R^) | This work |
| plasmid |  |  |
| POJ260 | Conjugal shuttle plasmid containing Apramycin resistance cassette; | Commercial |
| pSET152 | Integrative conjugal shuttle vector containing a constitutive *ermE* promoter for *Streptomyces spp.*; used for gene over-expression; apramycin resistance | Commercial |
| pPKS-2 | Construct for gene *AMK26_RS31425* (PKS-2, 126635-127240 bp) replaced by thiostrepton resistance gene; | This study |
| pPKS-3 | Construct for gene *AMK26_RS31915* (PKS-3, 235985-236846 bp) replaced by thiostrepton resistance gene; | This study |
| pPKS-4 | Construct for gene *AMK26_RS32125* (PKS-4, 278298-279900 bp) replaced by thiostrepton resistance gene; | This study |
| pPKS-8-1 | Construct for gene ***AMK26_RS33800/05/10*** (PKS-8**,** 246606-249396 bp) replaced by thiostrepton resistance gene; | This study |
| pPKS-8-2 | Construct for gene ***AMK26_RS33810*** (PKS-8**,** 248458-249220 bp) replaced by Kanamycin resistance gene; | This study |
| pTNL-15-1 | Construct for gene *AMK26_RS16100* (Terpene-15, 661198-663048 bp) replaced by thiostrepton resistance gene; | This study |
| pTNL-15-2 | Construct for gene *AMK26_RS16100* (Terpene-15, 661284-662907 bp) replaced by Kanamycin resistance gene; | This study |
| pPKS-17 | Construct for gene *AMK26_RS11740* (PKS-17, 712683-714489bp) replaced by thiostrepton resistance gene; | This study |
| pPKS-28 | Construct for gene *AMK26_RS03200* (PKS-28, 1049797-1051772 bp) replaced by thiostrepton resistance gene; | This study |
| p152-E | pSET152 harboring *tnmE*, for *tnmE* gene over-expression; apramycin resistance | This study |
| p152-E10 | pSET152 harboring *tnmE10*, for *tnmE10* gene over-expression; apramycin resistance | This study |
| p152-E3E4E5 | pSET152 harboring *tnmE3E4E5*, for *tnmE3E4E5* gene over-expression; apramycin resistance | This study |
| p152-H | pSET152 harboring *tnmH*, for *tnmH* gene over-expression; apramycin resistance | This study |
| p152-L | pSET152 harboring *tnmH*, for *tnmH* gene over-expression; apramycin resistance | This study |
| p152-B | pSET152 harboring *tnmB*, for *tnmB* gene over-expression; apramycin resistance | This study |
| p152-S3 | pSET152 harboring *tnmS3*, for *tnmS3* gene over-expression; apramycin resistance | This study |
| p152-T1 | pSET152 harboring *tnmT1*, for *tnmT1* gene over-expression; apramycin resistance | This study |

Apr^S^, apramycin sensitive; Tsr^S^, thiostrepton sensitive; Apr^R^, apramycin resistant; Tsr^R^, thiostrepton resistant；Kan^S^, kanamycin sensitive; Kan^R^, kanamycin resistant.

**Table S2:** Primers used in this study

| Primer | Sequence (5′ - 3′) | Function |
| --- | --- | --- |
| 2-up-R | ggccgcggatcctctagacacgccttgaggtagtccg | Gene replacement |
| 2-up-F | ttcgaatgtgaacagagcgtctcctcgatcgg | Gene replacement |
| 2-tsr-F | aggagacgctctgttcacattcgaacggtctct | Gene replacement |
| 2-tsr-R | aagtccaggtcgttatcggttggccgcgaga | Gene replacement |
| 2-down-F | gccaaccgataacgacctggacttctacatcatcca | Gene replacement |
| 2-down-R | acggccagtgccaagctttccagctcaccctccagg | Gene replacement |
| 2-F | acgcccaggaacttgctc | Verification of mutant strain |
| 2-R | ctgcggctgatggagaacac | Verification of mutant strain |
| 3-up-F | ggccgcggatcctctagaagggcgtacgtgaccagtg | Gene replacement |
| 3-up-R | ttcgaatgtgaacagccgaaaccgtccagccc | Gene replacement |
| 3-tsr-F | acggtttcggctgttcacattcgaacggtctctg | Gene replacement |
| 3-tsr-R | caggaccagttcttatcggttggccgcgaga | Gene replacement |
| 3-down-F | ggccaaccgataagaactggtcctgctgcgct | Gene replacement |
| 3-down-R | acggccagtgccaagcttgccgtggacggtacggatc | Gene replacement |
| 3-F | tactggaccggctgcacg | Verification of mutant strain |
| 3-R | gtctggtacgccctgctg | Verification of mutant strain |
| 4-up-F | ggccgcggatcctctagagttcgcgttcagcgtgtca | Gene replacement |
| 4-up-R | ttcgaatgtgaacactacgaggcgttcgtcacc | Gene replacement |
| 4-tsr-F | acgcctcgtagtgttcacattcgaacggtctctgc | Gene replacement |
| 4-tsr-R | ctggcccacattatcggttggccgcgagat | Gene replacement |
| 4-down-F | gccaaccgataatgtgggccaggtcgtagaag | Gene replacement |
| 4-down-R | acggccagtgccaagcttagacggcgagatccgagg | Gene replacement |
| 4-F | ctgccggtgctcgtctcc | Verification of mutant strain |
| 4-R | cagtacgtcgccccacagat | Verification of mutant strain |
| 8-1-up-F | ggccgcggatcctctagattcgcccgatgacgatcc | Gene replacement |
| 8-1-up-R | ttcgaatgtgaacagtgaggaggccccagtac | Gene replacement |
| 8-1-tsr-F | gtcaggggttcgtacttatcggttggccgcgag | Gene replacement |
| 8-1-tsr-R | gcctcctcactgttcacattcgaacggtctctg | Gene replacement |
| 8-1-down-F | cggccagtgccaagcttgtccagtaccagcccagg | Gene replacement |
| 8-1-down-R | acggccagtgccaagcttgaacccctgacatcagg | Gene replacement |
| 8-1-F | cctgatgtcaggggttcgtac | Verification of mutant strain |
| 8-1-R | gtactggggcctcctcac | Verification of mutant strain |
| 15-1-up-F | ggccgcggatcctctagactcctgcaccgggatctg | Gene replacement |
| 15-1-up-R | ttcgaatgtgaacagactgtcctccttctctagcg | Gene replacement |
| 15-1-tsr-F | aggaggacagtctgttcacattcgaacggtctct | Gene replacement |
| 15-1-tsr-R | cacgtccgcttatcggttggccgcgaga | Gene replacement |
| 15-1-down-F | ccaaccgataagcggacgtggtggtctgc | Gene replacement |
| 15-1-down-R | cggccagtgccaagcttccactggtaccggcaggt | Gene replacement |
| 15-1-F | ccggttcttctccagcagg | Verification of mutant strain |
| 15-1-R | ctcaccactcagcgtgtgat | Verification of mutant strain |
| 17-up-F | ggccgcggatcctctagaactccttcaagggcatggg | Gene replacement |
| 17-up-R | ttcgaatgtgaacatcgaggaccgtctcccaca | Gene replacement |
| 17-tsr-F | acggtcctcgatgttcacattcgaacggtctctg | Gene replacement |
| 17-tsr-R | ccatggccgacttatcggttggccgcgaga | Gene replacement |
| 17-down-F | caaccgataagtcggccatggcgtacga | Gene replacement |
| 17-down-R | cggccagtgccaagctttgtggccgatgcccttgc | Gene replacement |
| 17-F | tggccggggaacagcagg | Verification of mutant strain |
| 17-R | ggagtcacgatgagcgacgc | Verification of mutant strain |
| 28-up-F | ggccgcggatcctctagatacgccgaccgcaagaag | Gene replacement |
| 28-up-R | ttcgaatgtgaacattgcgccagaactcctcgac | Gene replacement |
| 28-tsr-F | agttctggcgcaatgttcacattcgaacggtctctg | Gene replacement |
| 28-tsr-R | gcccgatcatcttatcggttggccgcgaga | Gene replacement |
| 28-down-F | gccaaccgataagatgatcgggcacagcctc | Gene replacement |
| 28-down-R | cggccagtgccaagctttcgatggtgtggaacgtcct | Gene replacement |
| 28-F | ggcgacgaagagctcgacg | Verification of mutant strain |
| 28-R | cgacgtactcgccgaggc | Verification of mutant strain |
| 8-2-up-F | ggccagtgccaagcttgcggatgaccgtgctgtg | Gene replacement |
| 8-2-up-R | ttacgagttcttctgaaagatgaacgaccgccatgag | Gene replacement |
| 8-2-kana-F | cggtcgttcatctttcagaagaactcgtcaagaaggcg | Gene replacement |
| 8-2-kana-R | ccgaaggccctgtggaatgtgtgtcagttaggg | Gene replacement |
| 8-2-down-F | cggccagtgccaagcttgacctcaaggtcgctgcacc | Gene replacement |
| 8-2-down-R | cacattccacagggccttcgggtcccagtc | Gene replacement |
| 8-2-F | atggagctcaccggtgtg | Verification of mutant strain |
| 8-2-R | agtgggcgtcaaggagtact | Verification of mutant strain |
| 15-2-up-F | ggccagtgccaagcttgatgtgcgaggtgtgggc | Gene replacement |
| 15-2-up-R | gacgagttcttctgattcgcctattacgtggacggc | Gene replacement |
| 15-2-kana-F | cgtaataggcgaatcagaagaactcgtcaagaaggcg | Gene replacement |
| 15-2-kana-R | gttcgaggtgctgtggaatgtgtgtcagttaggg | Gene replacement |
| 15-2-down-F | gacgagttcttctgattcgcctattacgtggacggc | Gene replacement |
| 15-2-down-R | cggccagtgccaagcttgatgtgcgaggtgtgggc | Gene replacement |
| 15-2-F | atggagctcaccggtgtg | Verification of mutant strain |
| 15-2-R | agtgggcgtcaaggagtact | Verification of mutant strain |
| E-F | ccgatatccatatgggtgagcctgcccgtgac | Gene expression |
| E-R | caggtcgactctagagtgaccagggcgaggccg | Gene expression |
| E345-F-1 | ccgatatccatatgcatgcgggacatcacccaata | Gene expression |
| E345-R-1 | caggtcgactctagagtctgccacagctggtgg | Gene expression |
| E10-F | ccgatatccatatgcgtcgccgtgtccctctg | Gene expression |
| E10-R | caggtcgactctagactcgggttcagcgcacgg | Gene expression |
| S3-F | ccgatatccatatgtcggcgcccatggcaatc | Gene expression |
| S3-R | caggtcgactctagagaggtcaaccggccgac | Gene expression |
| L-F | ccgatatccatatggtcaccgcgtacctcgtc | Gene expression |
| L-R | caggtcgactctagattcacgtccggggtgagaac | Gene expression |
| B-F | ccgatatccatatgatgtcggaccaccacggc | Gene expression |
| B-R | caggtcgactctagagcatcagtcggtgccgcc | Gene expression |
| T1-F | ccgatatccatatgtggtccatgaacggaaacafa | Gene expression |
| T1-R | caggtcgactctagatgctcaggccgcggcgtc | Gene expression |
| E-E10-F | ccgatatccatatgggtgagcctgcccgtgac | Gene expression |
| E-E10-R | caggtcgactctagatcgtgcggctcgggttcag | Gene expression |
| H-F | ccgatatccatatgccgaccggcaatccgttc | Gene expression |
| H-R | caggtcgactctagaggccgttgggacgcgcag | Gene expression |

F stands for forward primer, and R stands for reverse primer. Red labeled letters were the overlapping regions designed for seamless cloning.

**Table S3:** Analysis of secondary metabolite BGCs in *S*. sp. CB03234 and CB03234-S

| BGC | Type | BGC | Type |
| --- | --- | --- | --- |
| 1 | Thiomitides | 21 | Lantipeptide |
| 2 | Type III PKS | 22 | Linaridin |
| 3 | Type III PKS | 23 | Lanthipeptide |
| 4 | Type I PKS | 24 | CDPS |
| 5 | *tnm* | 25 | Siderophore |
| 6 | NRPS | 26 | Phosphonate |
| 7 | NRPS | 27 | Terpene |
| 8 | Type II PKS | 28 | Type I PKS-NRPS |
| 9 | Terpene | 29 | Lanthipeptide |
| 10 | RiPP | 30 | Ectoine |
| 11 | NRPS | 31 | Lassopeptide |
| 12 | Lassopeptide | 32 | NRPS |
| 13 | Siderophore | 33 | PKS (I)-Terpene |
| 14 | RiPP | 34 | Lantipeptide |
| 15 | Terpene (*tnl*) | 35 | Lantipeptide |
| 16 | Siderophore | 36 | Amglyccycl |
| 17 | Type I PKS | 37 | Terpene |
| 18 | NRPS | 38 | PKS (I) |
| 19 | Terpene | 39 | PKS (II) |
| 20 | Melanin |  |  |

Note: red represents the *tnm* BGC, blue represents the *tnl* BGC, and green represents the missing fragment in CB03234-S.

**Table S4:** Knockout target genes and functions in biosynthetic gene clusters.

| BGC | ORFs (size) | Inactive gene | Function |
| --- | --- | --- | --- |
| 2-PKS (III) | AMK26_RS31415-31445 (7911bp) | AMK26_RS31425 | Type III polyketide synthase |
| 3-PKS (III) | AMK26_RS31910-31940 (7124bp) | AMK26_RS31915 | Type III polyketide synthase |
| 4-PKS (I) | AMK26_RS32045-32180 (43968bp) | AMK26_RS32125 | Type I polyketide synthase |
| 8-PKS (II) | AMK26_RS33780-34060 (61193bp) | **AMK26_RS33800/05/10** | **KS_α_/KS_β_/ACP** |
| 15- TNL | AMK26_RS16055-16150 (22565bp) | AMK26_RS16100 | **Diterpene synthase** |
| 17-PKS (I) | AMK26_RS11675-11815 (46675bp) | AMK26_RS11740 | Type I polyketide synthase |
| 28-PKS (I) | AMK26_RS03160-03355 (52800bp) | AMK26_RS03200 | Type I polyketide synthase |

**Table S5:** ^1^H and ^13^C NMR Spectroscopic Data (600 MHz, DMSO-*d*_6_) for TNM-W

| Position | *δ*_C_ | *δ*_H_ (*J* in Hz) |
| --- | --- | --- |
| 1 | NH | 10.19 (d, *J* = 4.4 Hz, 1H) |
| 2 | 143.43 |  |
| 3 | 109.85 |  |
| 4 | 187.56 |  |
| 5 | 117.25 |  |
| 6 | 152.21 |  |
| 7 | 154.08 |  |
| 8 | 116.60 | 7.42 (d, *J* = 8.5 Hz, 1H) |
| 9 | 120.05 | 7.77 (d, *J* = 8.5 Hz, 1H) |
| 10 | 124.81 |  |
| 11 | 184.93 |  |
| 12 | 111.99 |  |
| 13 | 156.61 |  |
| 14 | 126.71 | 8.21 (s, 1H) |
| 15 | 132.31 |  |
| 16 | 124.92 |  |
| 17 | 62.39 | 6.41 (d, *J* = 4.4 Hz, 1H) |
| 18 | 98.21 |  |
| 19 | 91.14 |  |
| 20 | 124.00 | 6.04 (d, *J* = 9.5 Hz, 1H) |
| 21 | 123.56 | 6.00 (d, *J* = 9.5 Hz, 1H) |
| 22 | 92.77 |  |
| 23 | 102.90 |  |
| 24 | 46.33 | 5.34 (d, *J* = 4.0 Hz, 1H) |
| 25 | 138.41 |  |
| 26 | 65.43 | 4.81 – 4.74 (m, 1H) |
| 27 | 24.98 | 1.33 (d, *J* = 6.4 Hz, 3H) |
| 7-OMe | 56.70 | 3.94 (s, 3H) |
| 6-OH | OH | 13.57 (s, 1H) |
| 13-OH | OH | 13.70 (s, 1H) |
| 17-OH | OH | 6.49 (d, *J* = 4.4 Hz, 1H) |
| 26-OH | OH | 5.48 (d, *J* = 4.0 Hz, 1H) |

**Table S6:** Predicted functions of ORFs in #2-BGC and their average values derived from transcriptome analysis.

| No. | Gene ID | Putative function | WT_fpkm | S_fpkm | log_2_ (S vs WT) | FDR | Significant |
| --- | --- | --- | --- | --- | --- | --- | --- |
| 1 | AMK26_RS31415 | metallophosphoesterase | 16.63 | 570.34 | 4.06 | 6.33E-34 | up |
| 2 | AMK26_RS31420 | acyl-CoA carboxylase subunit epsilon | 10.28 | 283.04 | 3.51 | 7.46E-10 | up |
| 3 | AMK26_RS31425 | type III polyketide synthase | 7.64 | 167.16 | 3.47 | 1.96E-32 | up |
| 4 | AMK26_RS31430 | cytochrome P450 | 15.07 | 358.01 | 3.54 | 1.19E-39 | up |
| 5 | AMK26_RS31435 | cupin | 25.53 | 358.19 | 2.80 | 2.22E-21 | up |
| 6 | AMK26_RS31440 | catalase HPII | 18.18 | 228.46 | 2.65 | 8.55E-30 | up |
| 7 | AMK26_RS31445 | MFS transporter | 9.40 | 324.35 | 4.08 | 9.52E-54 | up |

**Table S7:** Predicted functions of ORFs in #3-BGC and their average values derived from transcriptome analysis.

| No. | Gene ID | Putative function | WT_fpkm | S_fpkm | log_2_ (S vs WT) | FDR | Significant |
| --- | --- | --- | --- | --- | --- | --- | --- |
| 1 | AMK26_RS31910 | hypothetical protein | 13.58 | 640.29 | 4.50 | 9.42E-43 | up |
| 2 | AMK26_RS31915 | type III polyketide synthase | 12.41 | 566.27 | 4.48 | 3.53E-61 | up |
| 3 | AMK26_RS31920 | hypothetical protein | 24.67 | 410.93 | 3.03 | 2.16E-25 | up |
| 4 | AMK26_RS31925 | NAD(P)/FAD-dependent oxidoreductase | 16.51 | 636.24 | 4.25 | 6.77E-35 | up |
| 5 | AMK26_RS31930 | DUF2236 domain-containing protein | 38.20 | 471.21 | 2.57 | 1.98E-18 | up |
| 6 | AMK26_RS31935 | hypothetical protein | 27.59 | 510.23 | 3.25 | 2.86E-05 | up |
| 7 | AMK26_RS31940 | DoxX family protein | 16.25 | 562.46 | 4.16 | 2.48E-28 | up |

| No. | Gene ID | Putative function | WT_fpkm | S_fpkm | log_2_ (S vs WT) | FDR | Significant |
| --- | --- | --- | --- | --- | --- | --- | --- |
| 1 | AMK26_RS32045 | acyl-CoA dehydrogenase | 33.88 | 573.09 | 3.07 | 2.58E-36 | up |
| 2 | AMK26_RS32050 | acyl-CoA dehydrogenase | 33.03 | 351.58 | 2.39 | 2.66E-21 | up |
| 3 | AMK26_RS32055 | alcohol dehydrogenase | 25.55 | 393.39 | 2.93 | 7.91E-26 | up |
| 4 | AMK26_RS32060 | aminotransferase | 185.56 | 160.08 | -1.22 | 1.05E-05 | down |
| 5 | AMK26_RS32065 | PIG-L domain-containing protein | 68.61 | 338.86 | 1.28 | 4.14E-08 | up |
| 6 | AMK26_RS32070 | cellulase | 11.56 | 63.83 | 1.53 | 3.77E-06 | up |
| 7 | AMK26_RS32075 | acetylxylan esterase | 25.05 | 400.36 | 2.98 | 2.00E-33 | up |
| 8 | AMK26_RS32080 | oxidoreductase | 37.22 | 435.34 | 2.55 | 1.94E-22 | up |
| 9 | AMK26_RS32085 | oxidoreductase | 20.91 | 320.71 | 2.91 | 1.40E-27 | up |
| 10 | AMK26_RS32090 | cupin | 47.89 | 393.16 | 2.04 | 1.29E-08 | up |
| 11 | AMK26_RS32095 | Type-2Aa cytolytic delta-endotoxin | 44.72 | 113.01 | 0.33 | 0.325311897 | no |
| 12 | AMK26_RS32100 | UDP-glucose 6-dehydrogenase | 11.36 | 340.81 | 3.87 | 1.99E-53 | up |
| 13 | AMK26_RS32105 | hypothetical protein | 218.29 | 83.15 | -2.46 | 1.32E-10 | down |
| 14 | AMK26_RS32110 | hypothetical protein | 152.63 | 511.65 | 0.74 | 0.009493764 | no |
| 15 | AMK26_RS32115 | hydroxylase | 82.43 | 210.86 | 0.34 | 0.21652614 | no |
| 16 | AMK26_RS32120 | cytochrome P450 | 6.67 | 391.49 | 4.84 | 6.11E-65 | up |
| 17 | AMK26_RS32125 | type I polyketide synthase | 8.46 | 427.18 | 4.66 | 1.92E-46 | up |
| 18 | AMK26_RS32130 | 3-oxoacyl-ACP synthase | 10.52 | 278.34 | 3.70 | 7.39E-42 | up |
| 19 | AMK26_RS32135 | hypothetical protein | 12.28 | 591.90 | 4.56 | 7.60E-32 | up |
| 20 | AMK26_RS32140 | alpha/beta hydrolase | 14.02 | 75.13 | 1.65 | 1.01E-07 | up |
| 21 | AMK26_RS32145 | hypothetical protein | 21.34 | 461.60 | 3.46 | 3.09E-29 | up |
| 22 | AMK26_RS32150 | hypothetical protein | 13.74 | 473.55 | 4.09 | 1.17E-39 | up |
| 23 | AMK26_RS32155 | hypothetical protein | 28.35 | 230.21 | -4.75 | 3.31E-27 | down |
| 24 | AMK26_RS32160 | DNA-binding protein | 39.61 | 543.94 | -0.54 | 0.080988865 | no |
| 25 | AMK26_RS32165 | hypothetical protein | 92.07 | 381.98 | 1.03 | 2.56E-06 | up |
| 26 | AMK26_RS32170 | glyoxalase | 40.60 | 63.39 | -0.43 | 0.397556942 | no |
| 27 | AMK26_RS32175 | hypothetical protein | 79.21 | 501.86 | 2.79 | 1.43E-28 | up |
| 28 | AMK26_RS32180 | hypothetical protein | 5.92 | 439.62 | 5.21 | 7.85E-57 | up |
| 29 | AMK26_RS32185 | SAM-dependent methyltransferase | 10.30 | 517.07 | 4.66 | 3.71E-48 | up |

**Table S8:** Predicted functions of ORFs in #4-BGC and their average values derived from transcriptome analysis.

**Table S9:** Predicted functions of ORFs in #5 (TNM)-BGC and their average values derived from transcriptome analysis.

| Gene | Gene ID | Putative function | WT_fpkm | S_fpkm | log_2_ (S vs WT) | FDR | Significant |
| --- | --- | --- | --- | --- | --- | --- | --- |
| *tnmE6* | AMK26_RS32490 | flavin reductase | 153.05 | 388.38 | 0.92 | 2.88E-17 | up |
| *tnmR7* | AMK26_RS32495 | AraC family transcriptional regulator | 149.49 | 735.26 | 2.26 | 0.047390404 | up |
| *tnmB* | AMK26_RS32500 | enediyne self-sacrifice resistance protein | 1623.81 | 2512.35 | 1.17 | 0.036881227 | up |
| *tnmR3* | AMK26_RS32505 | Activator of bc1 complex (ABC) kinases | 214.72 | 426.86 | 1.18 | 1.28E-05 | up |
| *tnmC* | AMK26_RS32510 | ketone reductase | 100.85 | 208.69 | 1.28 | 2.42E-11 | up |
| *tnmD* | AMK26_RS32515 | PBS lyase HEAT-like repeat protein | 14892.13 | 15092.52 | 0.04 | 3.40E-16 | no |
| *tnmF* | AMK26_RS32520 | hypothetical protein | 34696.32 | 177907.43 | 3.93 | 6.12E-24 | up |
| *tnmG* | AMK26_RS32535 | S-adenosylmethionine-dependent methyltransferases | 2117.57 | 9222.83 | 4.63 | 5.81E-38 | up |
| *tnmH* | AMK26_RS32540 | O-methyltransferase | 1007.57 | 3877.59 | 2.20 | 2.94E-07 | up |
| *tnmE10* | AMK26_RS32545 | type II thioesterase | 213.30 | 1128.28 | 3.32 | 3.20E-23 | up |
| *tnmE* | AMK26_RS32550 | enediyne polyketie synthase | 85.60 | 937.49 | 2.59 | 5.79E-09 | up |
| *tnmE5* | AMK26_RS32555 | hypothetical protein | 74.30 | 1189.65 | 3.91 | 3.69E-20 | up |
| *tnmE4* | AMK26_RS32560 | hypothetical protein | 58.51 | 647.42 | 2.68 | 3.72E-06 | up |
| *tnmE3* | AMK26_RS32565 | hypothetical protein | 892.35 | 4323.48 | 3.94 | 3.69E-20 | up |
| *tnmI* | AMK26_RS32570 | oxidoreductase | 356.33 | 400.33 | 2.95 | 0.001065763 | up |
| *tnmR1* | AMK26_RS32575 | HxlR family transcriptional regulator | 3917.68 | 8596.78 | 1.29 | 1.32E-20 | up |
| *tnmJ* | AMK26_RS32580 | SAM-dependent methyltransferase | 944.54 | 1672.36 | 1.76 | 1.91E-07 | up |
| *tnmK1* | AMK26_RS32585 | alpha/beta hydrolases | 603.22 | 746.62 | 0.87 | 0.041972889 | no |
| *tnmK2* | AMK26_RS32590 | alpha/beta hydrolases | 579.47 | 741.96 | 0.74 | 0.683768992 | no |
| *tnmL* | AMK26_RS32595 | cytochrome P450 monooxygenase | 1123.86 | 1814.49 | 0.32 | 0.762757272 | no |
| *tnmM1* | AMK26_RS32600 | rieske (2Fe-2S) iron-sulfur domain protein | 191.56 | 310.32 | 0.19 | 0.602984805 | no |
| *tnmM2* | AMK26_RS32605 | rieske (2Fe-2S) iron-sulfur domain protein | 243.04 | 457.53 | 0.69 | 0.004847561 | no |
| *tnmN* | AMK26_RS32610 | SnoaL-like polyketide cyclase | 2585.81 | 3720.28 | 3.05 | 8.39E-36 | up |
| *tnmO* | AMK26_RS32615 | hypothetical protein | 4378.07 | 10921.11 | 2.70 | 3.47E-33 | up |
| *tnmP* | AMK26_RS32620 | epoxidase | 1398.82 | 2125.40 | 1.72 | 8.14E-11 | up |
| *tnmR2* | AMK26_RS32625 | putative tegulator | 758.97 | 1027.50 | 2.88 | 8.65E-33 | up |
| *tnmQ* | AMK26_RS32630 | hypothetical protein | 8316.67 | 6354.34 | 0.46 | 0.351691798 | no |
| *tnmS1* | AMK26_RS32635 | glyoxalase/bleomycin resisance protein/dioxygenase | 9061.22 | 3554.62 | -2.23 | 4.25E-18 | down |
| *tnmT1* | AMK26_RS32640 | Fungal trichothecene efflux pump (TRI12) | 5036.20 | 8376.07 | 1.73 | 1.86E-11 | up |
| *tnmS2* | AMK26_RS32645 | glyoxalase/bleomycin resisance protein/dioxygenase | 9838.87 | 15886.63 | 1.39 | 3.59E-08 | up |
| *tnmR4* | AMK26_RS32650 | AraC family transcriptional regulator | 295.97 | 394.98 | 1.68 | 1.86E-11 | up |
| *tnmT2* | AMK26_RS32655 | MFS transporter | 215.10 | 915.45 | 1.45 | 1.42E-09 | up |
| *tnmS3* | AMK26_RS32490 | glyoxalase/bleomycin resisance protein/dioxygenase | 11.52 | 353.98 | 3.63 | 6.06E-37 | up |

**Table S10:** Predicted functions of ORFs in #8-BGC and their average values derived from transcriptome analysis.

| No. | Gene ID | Putative function | WT_fpkm | S_fpkm | log_2_ (S vs WT) | FDR | Significant |
| --- | --- | --- | --- | --- | --- | --- | --- |
| 1 | AMK26_RS33780 | DNA-binding transcriptional regulator | 38.10 | 542.99 | 2.82 | 3.90E-34 | up |
| 2 | AMK26_RS33785 | NmrA family protein | 80.06 | 501.91 | 1.63 | 1.75E-13 | up |
| 3 | AMK26_RS33790 | hypothetical protein | 57.25 | 1421.56 | 3.44 | 1.04E-07 | up |
| 4 | AMK26_RS33795 | hypothetical protein | 12.11 | 566.41 | 4.54 | 3.53E-58 | up |
| 5 | AMK26_RS33800 | acyl carrier protein | 17.93 | 40.86 | 3.54 | 0.360024414 | no |
| 6 | AMK26_RS33805 | ketosynthase chain-length factor | 10.03 | 350.76 | 4.13 | 1.85E-55 | up |
| 7 | AMK26_RS33810 | beta-ACP synthase | 10.30 | 354.02 | 4.05 | 4.86E-47 | up |
| 8 | AMK26_RS33815 | methylmalonyl-CoA carboxyltransferase | 10.91 | 226.97 | 3.32 | 4.71E-32 | up |
| 9 | AMK26_RS33820 | AfsR/SARP family transcriptional regulator | 251.49 | 436.49 | -0.24 | 0.37913782 | no |
| 10 | AMK26_RS33825 | hypothetical protein | 41.33 | 23.24 | -1.72 | 0.006008486 | down |
| 11 | AMK26_RS33830 | hypothetical protein | 53.93 | 251.12 | 1.21 | 1.08E-05 | up |
| 12 | AMK26_RS33835 | sensor histidine kinase | 407.92 | 180.66 | -2.19 | 5.32E-13 | down |
| 13 | AMK26_RS33840 | LuxR family DNA-binding response regulator | 270.03 | 398.52 | -0.49 | 0.075851623 | no |
| 14 | AMK26_RS33850 | hypothetical protein | 25.49 | 362.05 | 2.80 | 1.97E-31 | up |
| 15 | AMK26_RS33855 | hypothetical protein | 12.88 | 426.01 | 4.04 | 8.91E-51 | up |
| 16 | AMK26_RS33860 | polyketide synthase | 12.16 | 290.64 | 3.59 | 1.52E-47 | up |
| 17 | AMK26_RS33870 | hypothetical protein | 137.19 | 120.06 | -1.21 | 3.95E-06 | down |
| 18 | AMK26_RS33875 | cyclase | 23.64 | 225.63 | 2.26 | 2.22E-18 | up |
| 19 | AMK26_RS33880 | hypothetical protein | 7.72 | 336.56 | 4.31 | 1.06E-39 | up |
| 20 | AMK26_RS33885 | glucose-1-phosphate thymidylyltransferase | 15.08 | 275.51 | 3.18 | 4.12E-37 | up |
| 21 | AMK26_RS33890 | dTDP-glucose 4,6-dehydratase | 8.03 | 127.88 | 2.95 | 3.32E-23 | up |
| 22 | AMK26_RS33895 | hypothetical protein | 20.47 | 330.69 | 2.99 | 1.32E-26 | up |
| 23 | AMK26_RS33900 | hypothetical protein | 13.33 | 16.80 | -0.67 | 0.399178317 | no |
| 24 | AMK26_RS33905 | hypothetical protein | 11.47 | 249.62 | 3.41 | 4.70E-39 | up |
| 25 | AMK26_RS33910 | dTDP-4-dehydrorhamnose 3,5-epimerase | 2.94 | 66.81 | 3.28 | 3.21E-11 | up |
| 26 | AMK26_RS33915 | lipopolysaccharide biosynthesis protein RfbH | 4.89 | 169.94 | 4.05 | 7.61E-41 | up |
| 27 | AMK26_RS33920 | hypothetical protein | 6.79 | 493.69 | 5.09 | 7.93E-58 | up |
| 28 | AMK26_RS33925 | cytochrome P450 | 7.79 | 189.03 | 3.63 | 6.58E-38 | up |
| 29 | AMK26_RS33930 | cytochrome | 31.34 | 203.60 | 4.56 | 0.214605125 | no |
| 30 | AMK26_RS33935 | hypothetical protein | 54.43 | 144.39 | 0.40 | 0.108186428 | no |
| 31 | AMK26_RS33940 | excinuclease ABC subunit A | 16.09 | 236.15 | 2.87 | 1.02E-30 | up |
| 32 | AMK26_RS33945 | cytochrome P450 | 4.21 | 88.33 | 3.45 | 5.92E-21 | up |
| 33 | AMK26_RS33950 | carnitine dehydratase | 11.56 | 481.22 | 4.36 | 2.96E-64 | up |
| 34 | AMK26_RS33955 | 3-oxoacyl-ACP synthase | 7.03 | 342.15 | 4.61 | 9.84E-58 | up |
| 35 | AMK26_RS33960 | alpha/beta hydrolase | 18.71 | 447.24 | 3.55 | 2.93E-42 | up |
| 36 | AMK26_RS33965 | ferredoxin | 8.47 | 139.84 | 2.41 | 0.000432675 | up |
| 37 | AMK26_RS33970 | hypothetical protein | 8.63 | 463.40 | 4.67 | 2.41E-50 | up |
| 38 | AMK26_RS33975 | hypothetical protein | 8.17 | 454.89 | 4.73 | 1.32E-61 | up |
| 39 | AMK26_RS33980 | NADP-dependent oxidoreductase | 7.63 | 324.23 | 4.40 | 1.89E-54 | up |
| 40 | AMK26_RS33985 | hypothetical protein | 7.33 | 172.74 | 3.76 | 3.44E-20 | up |
| 41 | AMK26_RS33990 | 4-hydroxybenzoate 3-monooxygenase | 7.09 | 349.40 | 3.55 | 4.05E-37 | up |
| 42 | AMK26_RS33995 | hypothetical protein | 12.34 | 137.53 | 3.75 | 1.21E-34 | up |
| 43 | AMK26_RS34000 | phospho-2-dehydro-3-deoxyheptonate aldolase | 7.16 | 102.47 | 3.21 | 3.76E-28 | up |
| 44 | AMK26_RS34005 | hypothetical protein | 8.15 | 208.85 | 2.59 | 1.17E-09 | up |
| 45 | AMK26_RS34010 | 5-carboxymethyl-2-hydroxymuconate semialdehyde dehydrogenase | 10.95 | 196.53 | 3.23 | 1.83E-39 | up |
| 46 | AMK26_RS34015 | 4-oxalocrotonate tautomerase | 12.44 | 237.71 | 3.20 | 0.360024414 | no |
| 47 | AMK26_RS34020 | 4-oxalocrotonate decarboxylase | 5.70 | 351.87 | 3.30 | 5.10E-24 | up |
| 48 | AMK26_RS34025 | hypothetical protein | 12.89 | 217.08 | 4.26 | 7.24E-06 | up |
| 49 | AMK26_RS34030 | hypothetical protein | 15.65 | 262.72 | 3.11 | 2.84E-25 | up |
| 50 | AMK26_RS34035 | hypothetical protein | 9.09 | 173.58 | 3.03 | 1.86E-31 | up |
| 51 | AMK26_RS34040 | NDP-hexose 2,3-dehydratase | 9.96 | 195.97 | 3.24 | 1.52E-35 | up |
| 52 | AMK26_RS34045 | hypothetical protein | 66.73 | 33.65 | 3.24 | 3.47E-27 | up |
| 53 | AMK26_RS34050 | hypothetical protein | 5.02 | 104.07 | -2.04 | 1.27E-08 | down |
| 54 | AMK26_RS34055 | ketoacyl reductase | 12.44 | 152.12 | 3.41 | 2.17E-16 | up |
| 55 | AMK26_RS34060 | hypothetical protein | 7.26 | 254.34 | 3.78 | 2.76E-11 | up |
| 56 | AMK26_RS34065 | MBL fold metallo-hydrolase | 7.09 | 172.74 | 4.11 | 1.44E-43 | up |

| No. | Gene ID | Putative function | WT_fpkm | S_fpkm | log_2_ (S vs WT) | FDR | Significant |
| --- | --- | --- | --- | --- | --- | --- | --- |
| 1 | AMK26_RS16055 | Tryptophan dioxygenase | 184.84 | 168.965 | -1.15 | 1.12E-05 | down |
| 2 | AMK26_RS16060 | Alpha beta hydrolase | 182.14 | 118.515 | -1.64 | 8.89E-12 | down |
| 3 | AMK26_RS16065 | cytochrome P450 | 132.52 | 106.955 | -1.31 | 8.11E-09 | down |
| 4 | AMK26_RS16070 | Peptidyl carrier protein | 327.93 | 136.25 | -2.41 | 2.97E-15 | down |
| 5 | AMK26_RS16075 | AMP-dependent synthetase | 163.61 | 120.255 | -1.45 | 3.57E-07 | down |
| 6 | AMK26_RS16080 | cytochrome P450 | 102.76 | 175.395 | -0.24 | 0.350875356 | no |
| 7 | AMK26_RS16085 | Fumarylacetocetate-like hydrolase | 101.40 | 189.675 | -0.12 | 0.625092445 | no |
| 8 | AMK26_RS16090 | Polyprenyl synthase | 118.96 | 112.75 | -1.08 | 2.16E-06 | down |
| 9 | AMK26_RS16095 | UbiA-type prenyltransferase | 86.67 | 244.385 | 0.47 | 0.044541757 | no |
| 10 | AMK26_RS16100 | Type Ⅱ diterpene synthase | 84.89 | 278.82 | 0.70 | 0.011355067 | no |
| 11 | AMK26_RS16105 | FAD-dependent GGPP epoxidase | 95.73 | 193.82 | 0.01 | 0.965490496 | no |
| 12 | AMK26_RS16110 | taurine dioxygenase | 218.13 | 72.655 | -2.60 | 2.43E-27 | down |
| 13 | AMK26_RS16115 | LuxR family regulator | 1378.00 | 158.58 | -4.36 | 1.32E-09 | down |
| 14 | AMK26_RS16120 | Na/H exchanger | 94.13 | 188.61 | 0.00 | 0.999118603 | no |
| 15 | AMK26_RS16125 | hypothetical protein | 120.79 | 100.405 | -1.28 | 4.56E-08 | down |
| 16 | AMK26_RS16130 | sodium-independent anion transporter | 38.51 | 46.475 | -0.72 | 0.024702063 | no |
| 17 | AMK26_RS16135 | MerR family transcriptional regulator | 138.29 | 338.95 | 3.08 | 3.53E-09 | up |
| 18 | AMK26_RS16140 | hypothetical protein | 15.42 | 269.705 | 3.04 | 3.18E-25 | up |
| 19 | AMK26_RS16145 | hypothetical protein | 37.38 | 307.485 | 2.04 | 1.73E-11 | up |
| 20 | AMK26_RS16150 | pyridine nucleotide-disulfide oxidoreductase | 184.84 | 168.965 | 3.41 | 3.53E-37 | up |

**Table S11:** Predicted functions of ORFs in #15 (TNL)-BGC and their average values derived from transcriptome analysis.

**Table S12:** Predicted functions of ORFs in #17-BGC and their average values derived from transcriptome analysis.

| No. | Gene ID | Putative function | WT_fpkm | S_fpkm | log_2_ (S vs WT) | FDR | Significant |
| --- | --- | --- | --- | --- | --- | --- | --- |
| 1 | AMK26_RS11675 | alpha-1,4-glucan--maltose-1-phosphate maltosyltransferase | 59.10 | 125.22 | 0.08 | 0.728238242 | no |
| 2 | AMK26_RS11680 | NAD-dependent epimerase/dehydratase | 24.50 | 214.78 | 2.10 | 6.02E-15 | up |
| 3 | AMK26_RS11685 | AraC family transcriptional regulator | 49.12 | 216.26 | 1.12 | 7.67E-07 | up |
| 4 | AMK26_RS11690 | 4-hydroxyphenylacetate 3-monooxygenase | 74.73 | 124.24 | -0.27 | 0.24915387 | no |
| 5 | AMK26_RS11695 | MFS transporter | 16.55 | 277.59 | 3.04 | 6.22E-33 | up |
| 6 | AMK26_RS11700 | monodechloroaminopyrrolnitrin synthase PrnB | 4.23 | 103.45 | 3.60 | 1.65E-27 | up |
| 7 | AMK26_RS11705 | flavin-dependent oxidoreductase | 10.31 | 238.63 | 3.49 | 7.24E-38 | up |
| 8 | AMK26_RS11710 | glycogen phosphorylase | 375.72 | 817.81 | 1.78 | 1.51E-08 | up |
| 9 | AMK26_RS11720 | hypothetical protein | 16.30 | 170.65 | 2.40 | 3.32E-19 | up |
| 10 | AMK26_RS11725 | hypothetical protein | 15.62 | 296.12 | 3.22 | 1.30E-35 | up |
| 11 | AMK26_RS11730 | hypothetical protein | 11.71 | 236.57 | 3.30 | 1.26E-39 | up |
| 12 | AMK26_RS11735 | enediyne biosynthesis protein UnbU | 10.16 | 140.34 | 2.78 | 1.71E-20 | up |
| 13 | AMK26_RS11740 | Beta-ketoacyl synthase | 13.53 | 278.52 | 3.36 | 1.32E-28 | up |
| 14 | AMK26_RS11745 | 4-hydroxybenzoyl-CoA thioesterase | 8.23 | 130.65 | 2.74 | 7.30E-09 | up |
| 15 | AMK26_RS11750 | flavin reductase | 26.68 | 233.53 | 2.05 | 7.15E-11 | up |
| 16 | AMK26_RS11755 | peptidase M4 | 305.43 | 817.52 | 2.38 | 8.19E-14 | up |
| 17 | AMK26_RS11760 | NAD(P)H-dependent oxidoreductase | 93.47 | 129.93 | -0.58 | 0.095749738 | no |
| 18 | AMK26_RS11765 | ABC transporter | 110.50 | 178.09 | -0.32 | 0.249720548 | no |
| 19 | AMK26_RS11770 | ABC transporter | 155.08 | 254.14 | -0.29 | 0.309597595 | no |
| 20 | AMK26_RS11775 | multidrug ABC transporter ATP-binding protein | 5.19 | 149.24 | 3.69 | 6.30E-26 | up |
| 21 | AMK26_RS11780 | hypothetical protein | 6.18 | 303.36 | 4.44 | 1.47E-31 | up |
| 22 | AMK26_RS11785 | sensor histidine kinase | 12.87 | 161.04 | 2.60 | 1.12E-23 | up |
| 23 | AMK26_RS11790 | DNA-binding response regulator | 58.11 | 227.71 | 0.95 | 0.000406878 | no |
| 24 | AMK26_RS11795 | glycogen debranching enzyme GlgX | 127.67 | 234.18 | -0.12 | 0.737169277 | no |
| 25 | AMK26_RS11800 | hypothetical protein | 44.06 | 150.32 | 0.77 | 0.000757012 | no |
| 26 | AMK26_RS11805 | hypothetical protein | 410.09 | 88.01 | -3.23 | 4.51E-26 | down |
| 27 | AMK26_RS11810 | enoyl-CoA hydratase | 469.26 | 224.61 | -2.09 | 3.10E-13 | down |
| 28 | AMK26_RS11815 | hypothetical protein | 2693.56 | 213.73 | -4.72 | 2.07E-40 | down |

**Table S13:** Predicted functions of ORFs in #28-BGC and their average values derived from transcriptome analysis.

| No. | Gene ID | Putative function | WT_fpkm | S_fpkm | log_2_ (S vs WT) | FDR | Significant |
| --- | --- | --- | --- | --- | --- | --- | --- |
| 1 | AMK26_RS03160 | SAM-dependent methyltransferase | 99.22 | 61.045 | -1.69 | 4.85E-12 | down |
| 2 | AMK26_RS03165 | SAM-dependent methyltransferase | 116.28 | 24.69 | -3.18 | 3.09E-21 | down |
| 3 | AMK26_RS03170 | hypothetical protein | 40.79 | 94.95 | 0.22 | 0.397721366 | no |
| 4 | AMK26_RS03175 | LLM class flavin-dependent oxidoreductase | 46.78 | 196.58 | 1.04 | 4.66E-05 | up |
| 5 | AMK26_RS03180 | oxidoreductase | 89.92 | 173.595 | -0.07 | 0.808537571 | no |
| 6 | AMK26_RS03185 | 3-oxoacyl-ACP synthase | 201.71 | 158.245 | -1.36 | 2.18E-07 | down |
| 7 | AMK26_RS03190 | acyl carrier protein | 173.78 | 43.145 | -3.29 | 0.000234423 | down |
| 8 | AMK26_RS03195 | 3-oxoacyl-ACP synthase | 71.18 | 180.485 | 0.32 | 0.22120117 | no |
| 9 | AMK26_RS03200 | SARP family transcriptional regulator | 65.78 | 116.11 | -0.23 | 0.423914013 | no |
| 10 | AMK26_RS03205 | hypothetical protein | 291.08 | 94.6 | -2.64 | 3.38E-21 | down |
| 11 | AMK26_RS03210 | non-ribosomal peptide synthetase | 192.98 | 163.89 | -1.23 | 0.000106295 | down |
| **12** | AMK26_RS03215 | flavin-dependent oxidoreductase | 262.02 | 172.975 | -1.60 | 4.76E-07 | down |
| 13 | AMK26_RS03220 | type I polyketide synthase | 285.18 | 151.715 | -1.91 | 1.67E-09 | down |
| 14 | AMK26_RS03225 | peptidase M38 | 362.23 | 73.985 | -3.29 | 1.12E-24 | down |
| 15 | AMK26_RS03230 | MbtH family protein | 364.19 | 174.095 | -5.32 | 0.03353054 | down |
| 16 | AMK26_RS03235 | FMN reductase | 408.66 | 154.01 | -2.45 | 6.33E-21 | down |
| 17 | AMK26_RS03240 | methylmalonyl-CoA carboxyltransferase | 302.79 | 67.23 | -3.17 | 2.25E-22 | down |
| 18 | AMK26_RS03245 | hypothetical protein | 257.59 | 681.1 | 0.45 | 0.734797477 | no |
| 19 | AMK26_RS03250 | hypothetical protein | 216.35 | 112.87 | -1.94 | 6.25E-11 | down |
| 20 | AMK26_RS03255 | aminobenzoate oxygenase | 1225.5 | 64.785 | -5.24 | 1.05E-44 | down |
| 21 | AMK26_RS03260 | hypothetical protein | 857.79 | 210.665 | -3.03 | 6.30E-18 | down |
| 22 | AMK26_RS03265 | hypothetical protein | 655.57 | 101.645 | -3.72 | 1.80E-30 | down |
| 23 | AMK26_RS03270 | ATP-binding protein | 83.82 | 175.195 | 0.05 | 0.835209629 | no |
| 24 | AMK26_RS03275 | Ohr subfamily peroxiredoxin | 32.73 | 201.95 | 1.52 | 3.33E-05 | up |
| 25 | AMK26_RS03280 | MarR family transcriptional regulator | 83.27 | 243.205 | 0.47 | 0.081602129 | no |
| 26 | AMK26_RS03285 | acetyl-CoA carboxylase | 57.47 | 289.895 | 1.31 | 5.82E-09 | up |
| 27 | AMK26_RS03290 | acyl-CoA synthetase | 64.13 | 242.12 | 0.90 | 4.60E-05 | no |
| 28 | AMK26_RS03295 | MFS transporter | 6.46 | 209.405 | 3.94 | 6.64E-42 | up |
| 29 | AMK26_RS03300 | histidine kinase | 13.56 | 260.42 | 3.23 | 4.00E-32 | up |
| 30 | AMK26_RS03305 | rod shape-determining protein | 33.14 | 199.975 | 1.57 | 1.16E-11 | up |
| 31 | AMK26_RS03310 | hypothetical protein | 28.12 | 339.42 | 2.54 | 4.07E-18 | up |
| 32 | AMK26_RS03315 | hypothetical protein | 54.31 | 287.15 | 1.36 | 1.02E-06 | up |
| 33 | AMK26_RS03320 | MarR family transcriptional regulator | 102.68 | 368.235 | 0.78 | 0.014427731 | no |
| 34 | AMK26_RS03325 | NADP-dependent oxidoreductase | 90.72 | 120.77 | -0.59 | 0.036899697 | no |
| 35 | AMK26_RS03330 | organic hydroperoxide resistance protein | 1431.00 | 120.84 | -4.73 | 7.15E-31 | down |
| 36 | AMK26_RS03335 | hypothetical protein | 84.62 | 192.85 | 0.18 | 0.538137827 | no |
| 37 | AMK26_RS03340 | lysine transporter LysE | 27.39 | 16.58 | -1.71 | 1.50E-05 | down |
| 38 | AMK26_RS03345 | diaminopimelate decarboxylase | 275.87 | 199.8 | -1.47 | 3.82E-07 | down |
| 39 | AMK26_RS03350 | pyridoxal-5'-phosphate-dependent protein subunit beta | 249.79 | 172.85 | -1.54 | 9.52E-08 | down |
| 40 | AMK26_RS03355 | cysteine desulfurase | 183.94 | 221.025 | -0.74 | 0.004958503 | no |

**Fig. S1.** Disruption of BGCs via PCR-targeting. (A) Schematic representation for disruption of BGC. (B) PCR analyses of the WT strain and the BGC double-cross mutant carried out using the primers listed in Table S2. S: CB03234-S; M: DL5000.

**A**


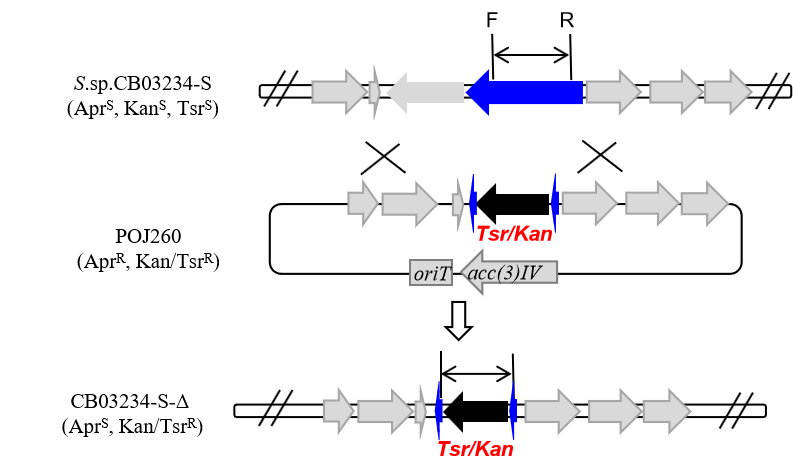


**B**


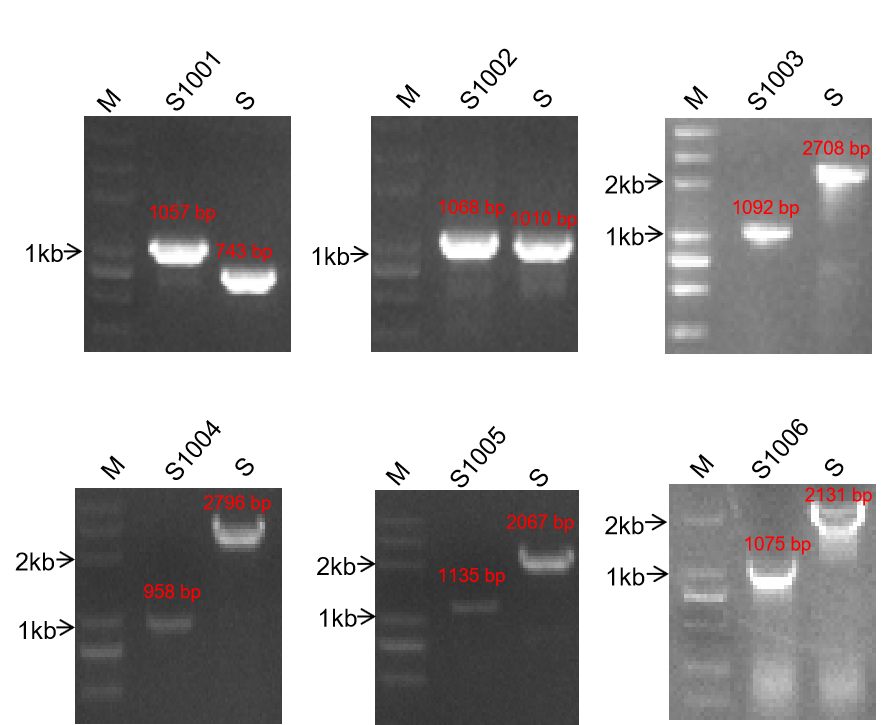


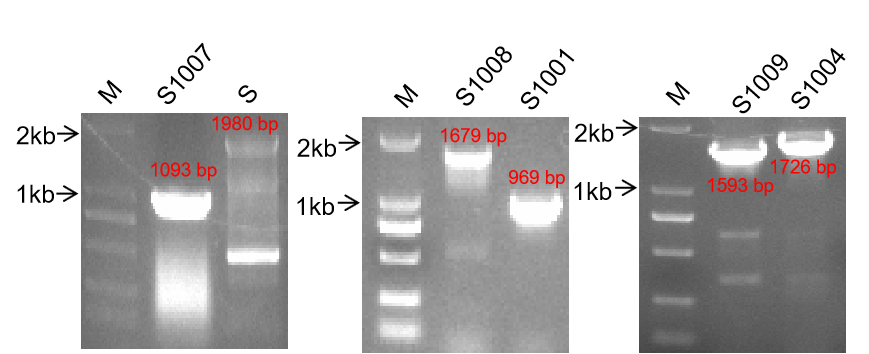


**Fig. S2.** The HPLC profiles of the CB03234-S and mutant strains: (A) In the resin, HPLC profiles of CB03234-S and mutant strains, S1004 (Δ#8-BGC), S1005 (Δ#15-BGC); (B) In the supernatant, HPLC profiles of CB03234-S and mutant strains, CB03234-S-*AMK26*_*RS31425*, (overexpression of the core pks gene (AMK26_ RS31425) from #2-BGC in CB03234-S).


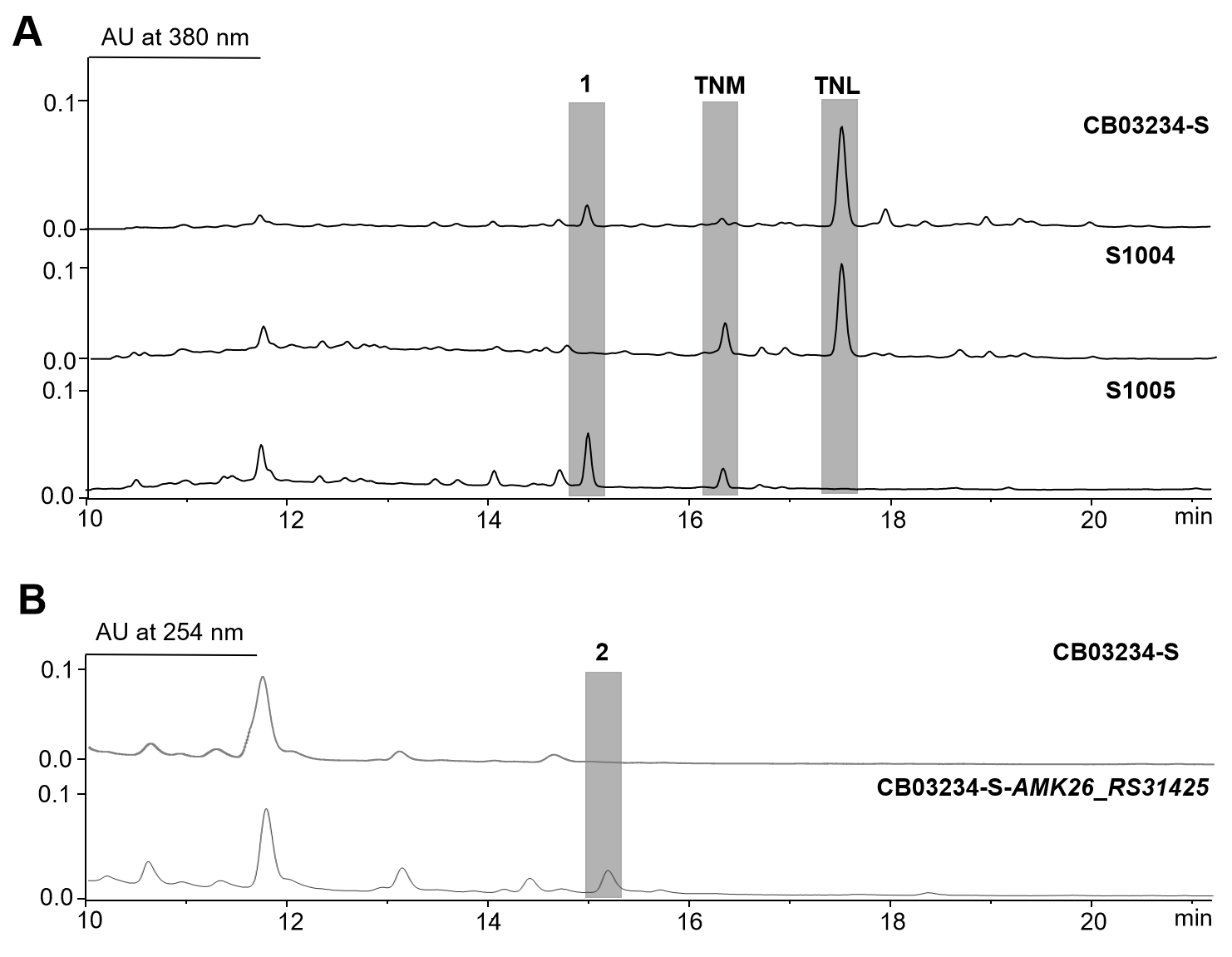


**Fig. S3.** The resistance of overexpression resistance genes mutants to TNM-A; S1016 (CB03234-S+*tnmT1*); S1017 (CB03234-S+*tnmB*); S1018 (CB03234-S+*tnmS3*).


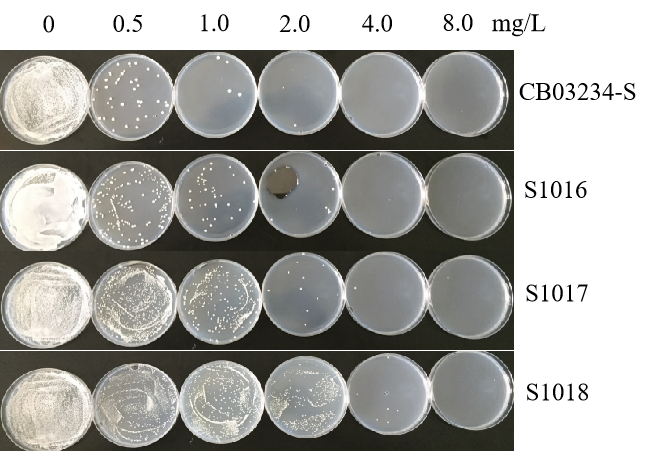


**Fig. S4.** Time course of TNM-W, TNM-A, and TNM-D production of S1021 strain in 30 L fermenter.

**Fig. S5.** HR-ESI (+) MS spectrum of TNM-W


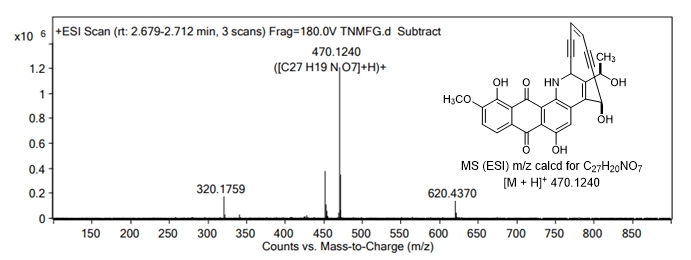


**Physicochemical Properties of TNM-W**

Purple powder; [α]_D_ ^25^ + 800.0 (C = 0.001, CH_3_OH); UV (CH_3_OH) λ_max_ (logε) 253 (4.32), 287 (shoulder), 318 (shoulder), 561 (3.79), 593 (3.66) nm; IR (neat) 3424, 2921, 1677, 1577, 1459, 1247, 728 cm^-1^.

**Fig. S6.** ^1^H NMR (600 MHz) spectrum of TNM-W in DMSO-*d*_6_

**Fig. S7**. ^13^C NMR (150 MHz) spectrum of TNM-W in DMSO-*d*_6_

**Fig. S8**. HSQC NMR (600 MHz) spectrum of TNM-W in DMSO-*d*_6_

**Fig. S9**. ^1^H -^1^H COSY NMR (600 MHz) spectrum of TNM-W in DMSO-*d*_6_

**Fig. S10**. HMBC NMR (600 MHz) spectrum of TNM-W in DMSO-*d*_6_

**Fig. S11**. NOESY NMR (600 MHz) spectrum of TNM-W in DMSO-*d*_6_

**References**
